# Image segmentation and separation of spectrally similar dyes in fluorescence microscopy by dynamic mode decomposition of photobleaching kinetics

**DOI:** 10.1186/s12859-022-04881-x

**Published:** 2022-08-12

**Authors:** Daniel Wüstner

**Affiliations:** Department of Biochemistry and Molecular Biology and Physics of Life Sciences (PhyLife) Center, University of Southern Denmark, Campusvej 55, DK-5230 Odense, Denmark

**Keywords:** Spatiotemporal modeling, Fluorescence, Photobleaching, Live-cell microscopy, Autofluorescence, Matrix methods

## Abstract

**Background:**

Image segmentation in fluorescence microscopy is often based on spectral separation of fluorescent probes (color-based segmentation) or on significant intensity differences in individual image regions (intensity-based segmentation). These approaches fail, if dye fluorescence shows large spectral overlap with other employed probes or with strong cellular autofluorescence.

**Results:**

Here, a novel model-free approach is presented which determines bleaching characteristics based on dynamic mode decomposition (DMD) and uses the inferred photobleaching kinetics to distinguish different probes or dye molecules from autofluorescence. DMD is a data-driven computational method for detecting and quantifying dynamic events in complex spatiotemporal data. Here, DMD is first used on synthetic image data and thereafter used to determine photobleaching characteristics of a fluorescent sterol probe, dehydroergosterol (DHE), compared to that of cellular autofluorescence in the nematode Caenorhabditis elegans. It is shown that decomposition of those dynamic modes allows for separating probe from autofluorescence without invoking a particular model for the bleaching process. In a second application, DMD of dye-specific photobleaching is used to separate two green-fluorescent dyes, an NBD-tagged sphingolipid and Alexa488-transferrin, thereby assigning them to different cellular compartments.

**Conclusions:**

Data-based decomposition of dynamic modes can be employed to analyze spatially varying photobleaching of fluorescent probes in cells and tissues for spatial and temporal image segmentation, discrimination of probe from autofluorescence and image denoising. The new method should find wide application in analysis of dynamic fluorescence imaging data.

**Supplementary Information:**

The online version contains supplementary material available at 10.1186/s12859-022-04881-x.

## Background

Image segmentation in fluorescence microscopy is an important image processing step to discriminate different image regions into spatially distinct sets. It is either based on differences in intensity of one fluorescent probe, e.g., DAPI intensity is selective for the nucleus but not found in other organelles, or on using distinct dyes for each subcellular compartment. Selective detection of different fluorescent molecules in intracellular organelles requires differences in emission wavelength (color) but this approach fails if spectral properties of dyes are very similar. Spectral unmixing can overcome this limitation to some extend but only, as long as the excitation or emission peaks of individual fluorophores differ by at least 30 nm [1]. Dye-specific properties, such as fluorescence lifetime can also be employed for selective separation of different probes or of probe from autofluorescence [2], but this requires special equipment not being available in many cell biological laboratories. Acquisition of spatio-temporal image data, i.e., videos, allows for including temporal information into the segmentation process. In principle, this should allow one to infer the contribution of several fluorophores or probe versus autofluorescence even in the same image region.

Photobleaching kinetics of, for example organelle markers, can be used as temporal information to segment intracellular organelles or to distinguish probe intensity from autofluorescence [3]. This is particularly important, if excitation and fluorescence spectra of a probe and of autofluorescence overlap strongly and can hardly be distinguished based on intensity differences. One such application is the analysis of sterol trafficking in the nematode Caenorhabditis elegans (*C. elegans*). *C. elegans* is a sterol-auxotroph organism, which is often employed to study the molecular basis of lipid transport and metabolic regulation on a systemic level [4–6]. Almost the entire sterol pool of this nematode can be replaced by feeding these worms with the fluorescent natural sterol dehydroergosterol (DHE), allowing for observing sterol uptake and transport by microscopy [7, 8]. To detect DHE selectively and to distinguish it from cellular autofluorescence in the ultraviolet, we made use of the much faster photobleaching of the sterol compared to autofluorescence of subcellular structures, particularly of gut granules [8, 9]. Pixel-wise fitting of a mathematical decay model to the bleaching kinetics allows for image segmentation and for detecting heterogeneous bleaching of probes in subcellular organelles [10, 11]. From such a model, other parameters, such as the integrated probe intensity can be inferred, and image background can be detected and corrected for [3, 10, 12, 13]. The success of model-based bleaching analysis for the above-described applications depends on accurate modeling of the bleaching process. As the underlying photophysics can be complex and is not directly accessible from first principles, particularly not for the heterogeneous intracellular environment, the mathematical decay models used to describe photobleaching must be considered as empirical fitting functions with some mechanistic underpinning [10, 14, 15]. To account for complex bleaching mechanisms, distributions of rate constants are often invoked in the modeling process, whose mechanistic interpretation is difficult [10, 11, 16, 17]. On the other hand, multi-exponential fitting suffers from the well-known non-orthogonality of real exponential functions, making that fluorescence decays cannot be uniquely represented by the sum of exponential functions with real exponents [18]. Moreover, the fit quality of any model depends on minimizing movement of the specimen, since displacement of for example organelles during imaging will reduce fitting accuracy. This can only be partly alleviated by temporal and/or spatial filtering techniques, which comes to the price of eventual loss of resolution. A recent study combined the analysis of photobleaching characteristics with spectral unmixing and employed a non-negative matrix factorization to separate several fluorescent probes with nearly identical emission spectra [19]. This study demonstrated the potential of including fluorophore bleaching characteristics into spectral separation methods for live-cell imaging. But it was limited by the fact that for each fluorescent probe, only a single bleaching fingerprint was considered. Many studies have shown, however, that photobleaching of fluorescent probes in complex environments like cells is heterogeneous [3, 11, 20–23]. In addition, uneven illumination and variations of refractive index can contribute to locally varying photobleaching kinetics in wide field and confocal microscopy [10, 12, 24, 25]. Together, this demands a full spatiotemporal description of the photobleaching characteristics for proper image segmentation and separation of spectrally indistinguishable fluorophores.

Dynamic mode decomposition (DMD) is a novel computational approach to extract dynamic information from large spatiotemporal datasets, such as images. DMD can be considered as a combination of principal component analysis (PCA) or singular value decomposition (SVD) with Fourier transformation in time [26, 27]. Originating in fluid mechanics as a method to determine coherent flow patterns, DMD is increasingly applied in computer vision and biomedical imaging [26, 28]. For example, DMD has been used to detect video shots or to separate foreground from background in image sequences [27, 29, 30]. It has also been used to segment images of kidneys and detect functional brain states by magnetic resonance imaging [31, 32]. Based on a spectral decomposition of the transfer or Koopman operator, DMD allows not only for detecting characteristic dynamic patterns in high-dimensional data sets, but also to dissect experimentally determined dynamics into individual dynamic modes. In this study, it is shown, how DMD can be employed to determine photobleaching characteristics of fluorescent probes and to distinguish probe fluorescence from cellular autofluorescence. This paper is organized as follows; first, the theory behind DMD is briefly reviewed, second, the method is applied to synthetic data of differently bleaching regions in simulated images. Third, an example of bleaching analysis in intact animals is given, where it is shown that the characteristic photobleaching of DHE can be detected by DMD and distinguished from cellular autofluorescence in *C. elegans*. Fourth, it is demonstrated that DMD of the distinct photobleaching kinetics of two green-emitting fluorescent probes can be used to distinguish them in living cells. Specifically, an NBD-tagged sphingolipid and Alexa488-tagged transferrin (Alexa488-Tf), an endocytosis marker, are employed, and DMD of their photobleaching kinetics allows for their unequivocal assignment to different subcellular organelles. Finally, the findings are discussed and brought into perspective for future applications of DMD in image analysis for life science applications.

## Results

### Theory of DMD applied to fluorescence photobleaching

**Fig. 1:**
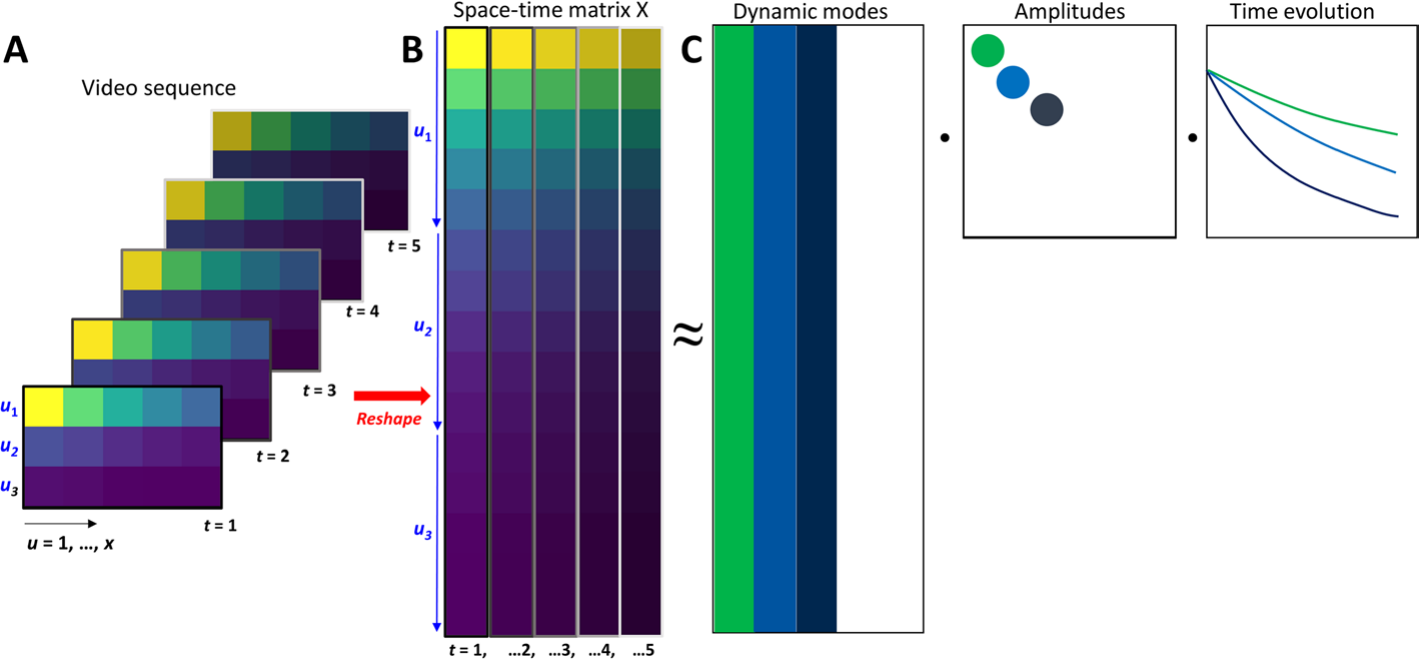
Workflow for dynamic mode decomposition of bleaching kinetics in fluorescence microscopy. **A** video sequence with decaying intensity *I*(*x*, *y*, *t*) in *m* frames representing time points *t* = 1, … m (A) gets first reshaped into a space–time matrix in which each video frame having *x* pixels in each row *u*_*y*_ (here *y* = 3) gets reshaped into *m* columns with *y* times *x* elements each of a space–time matrix *X* (**B** and see Eq. 1). Following the procedure described in Eq. 2–11 the system matrix describing the fluorescence dynamics gets dimensionally reduced using a SVD and rank truncation to capture the dominant dynamics in the system. Subsequently, spectral decomposition of this truncated system matrix provides the dynamic modes (eigenfunctions), mode amplitudes and eigenvalues, which together approximate the original space–time matrix *X* (**C**). Reconstruction of the video sequence is achieved by reversing the reshaping procedure described in panel A. In this example, a rank-3 approximation of the system matrix is illustrated. See text for further explanations

Let *I*(*x, y, t*) be a video image sequence of fluorescence intensity, I, as a function of spatial coordinates, *x, y* $\in$ *R*^n^ and time, *t* $\in$ *R*^m^. For the purpose of this study, this image sequence is supposed to contain the spatial distribution of some fluorescence probe in a living specimen, in which the fluorescence intensity decays over time due to photobleaching. Images have been acquired at discrete and constant time intervals, Δt, given by the microscope acquisition time. DMD is based on identifying the transfer operator between snapshots of the data measured at different instances of time, allowing one to determine the time evolution of a dynamic system. The time evolution considered here is the bleaching kinetics used for spatiotemporal segmentation of fluorescence images. In DMD, this is formulated as a matrix separation problem aimed to separate image regions with slowly or non-changing fluorescence intensity from regions experiencing more rapid photobleaching. To determine the low-rank structure of continuously varying fluorescence intensity in distinct image regions, the time-lapse image stack must first be reshaped into a space–time data matrix. Specifically, each image of dimension *x* times *y* is reshaped into a column vector (with *n* = x*∙y* elements), a so-called DMD snapshot, and the obtained vectors are stacked together over the *m* time points to create a single matrix of size *n* times *m* (i.e., a data matrix X $\in$ *R*^n∙m^. Figure 1A). Reshaping of each image into a column vector, $\overline{{x }_{k}}$, is carried out in a row-wise manner, i.e., all *x* pixels of the first row, *u*_1_ = (u_11_, u_12_, …, u_1x_) get stacked into the first *x* elements of the column vector, $\overline{{x }_{k}}$, followed by the next row of the image, *u*_2_ = (u_21_, u_22_, …, u_2x_) and so on, until *u*_y_ = (u_y1_, u_y2_, …, u_yx_). This is illustrated in Fig. 1A, B (blue symbols and arrows) for a 5 × 3 image size. Those image column vectors become arranged in an *n* × *m* data matrix X consisting of *m* columns, each representing one time point of the original image sequence [28, 31]:

1 $$X = \left[ {\overline{{x_{1} }} , \overline{{x_{2} }} , \cdots ,\overline{{x_{k} }} , \ldots \overline{{x_{m} }} } \right]$$

Here, the index *k* = 1,…, *m* indicates the frame number resembling the time axis of the video sequence. Note that this image reshaping procedure is entirely reversible (and only one line of code in NumPy [33]), such that location information between adjacent pixels is preserved. Having reshaped the image data, we want to find a mapping between discrete time steps Δt from state x(k∙Δt) = x_k_ to x_k+1_ as:

2 $$x_{k + 1} = A \cdot x_{k}$$

Here, *A* is a matrix which describes the advancement of the system in time and which resembles the Koopman or transfer operator for measurements g(*x*_k_) = *x*_k_ [26]. We want to approximate *A* solely from the given data. For that, we define the discrete time-shifted states of our system as two new matrices, X_1_, X_2_ $\in$ *R*^n∙(m−1)^:

3 $$X_{1} = \left[ {\overline{{x_{1} }} , \overline{{x_{2} }} , \cdots ,\overline{{x_{m - 1} }} } \right]$$

and

4 $$X_{2} = \left[ {\overline{{x_{2} }} , \overline{{x_{3} }} , \cdots ,\overline{{x_{m} }} } \right]$$

With that the system corresponding to Eq. (2) becomes *X*_2_ = *A*∙*X*_*1*_, from which we can find *A* by minimizing the Frobenius norm, $\left\| \cdot \right\|_{F}$ [26]:

5 $$A: = argmin\left\| {X_{2} - A \cdot X_{1} } \right\|_{F} = X_{2} \cdot X_{1}^{inv}$$

We can find the pseudoinverse of the first data matrix, $X_{1}^{inv}$, by using a SVD of *X*_1_ into unitary matrices U $\in$ *R*^n∙(m−1)^, V*$\in$ *R*^(m−1)∙(m−1)^ with singular values in the diagonal matrix Σ $\in$ *R*^(m−1)∙(m−1)^:

6 $$X_{{1}} = U \cdot \Sigma \cdot V^*$$

Since the data matrices, *X*_1_ and *X*_2_ have typically many more rows *n* (i.e., pixels for each image) than columns (*m*-1) (i.e., time points), there are at most (*m*-1) non-zero singular values and corresponding singular vectors and therefore, the matrix *A* will have at most rank (*m*-1), but in practice it is calculated up to rank *r* <  < (*m*-1). Thus, one can approximate *A* by calculating its projection onto the left singular vectors, i.e., the column vectors of U and truncating at rank *r* to only keep the governing dynamics of the system. We get using a similarity transformation [26, 29]:

7 $$A^{\prime} = U^{\prime*} \cdot A \cdot U^{\prime} = \, U^{\prime*} \cdot X_{2} \cdot V^{\prime} \cdot \Sigma ^{{\prime}{ - {1}}}$$

Here, *U*’, *V*’ and *Σ*’ are rank *r* approximations of the full matrices, *U*, *V* and *Σ*, and * indicates the complex conjugate transpose of a given matrix (which is the transpose for a real matrix). The similarity transformation of Eq. 7 reduces the size of the system matrix from *A* $\in$ *R*^(m−1)∙(m−1)^ to *A’*$\in$* R*^r∙r^ and resembles the dimension reduction used in PCA [26]. But in contrast to PCA, which is only used for spatial matrix decomposition, in DMD the system matrices *A* and *A’* contain spatial and temporal information. Having defined the reduced system matrix, one can solve the following eigenvalue problem to determine the eigenvalues describing the temporal evolution of the studied system by spectral decomposition of A’. First, one gets the eigenvalues from the matrix Λ:

8 $$A^{\prime} \cdot W = \Lambda \cdot W$$

Here, the matrix W contains the eigenvectors of *A*’, providing a coordinate transformation which diagonalizes *A*’ thereby decoupling the system dynamics. Importantly, these eigenvalues and the corresponding eigenfunctions collected in the matrix, *Φ*, are the same as for the full matrix *A* [26]:

9 $$\Phi = X_{{2}} \cdot V^{\prime} \cdot \Sigma ^{{\prime}{ - {1}}} \cdot W$$

Using this spectral decomposition of the transfer matrices A and A’, respectively, we can express the dynamics of our system as linear combination of eigenfunctions, φ_j_, (also called DMD modes or mode weights), corresponding eigenvalues, λ_j_ (so-called DMD eigenvalues) and mode amplitudes *b*, which are just the components of each eigenfunction in a given direction. This leads for discrete-time systems to:

10 $$x_{k} = \mathop \sum \limits_{j = 1}^{r} \varphi_{j} \cdot \lambda_{j}^{k - 1} \cdot b_{j}$$

And by using the continuous eigenvalues *ω* = ln(*λ*/Δ*t*), the Fourier modes, Eq. (10) can be written as [27]:

11 $$x\left( t \right) = \mathop \sum \limits_{j = 1}^{r} \varphi_{j} \cdot e^{{\omega_{j} \cdot t}} \cdot b_{j}$$

Here, x(t) is a vector of images (x, y index omitted for brevity) as a function of time, t, describing the time evolution of the entire system as sum of dynamic modes, *φ*_j_, mode amplitudes, *b*_j_, and time-dependent exponential functions (Fig. 1C). The eigenvalues, *ω*_j_, resemble the complex Fourier modes of the system where the real part describes the mode’s decay or increase, while the imaginary part describes mode oscillations. By this approach, the spatiotemporal image data can be decomposed in a purely data-driven manner, revealing dynamic properties of the system. In the following it is shown, how DMD can be used to discriminate differently bleaching fluorescent species in an image stack, first on synthetic data and then on experimental image stacks containing bleaching fluorophores and autofluorescence, respectively.

### DMD of synthetic bleach stacks

To assess the potential of DMD to capture the dynamics of photobleaching in microscopy images, synthetic image stacks with known bleach rates were used. A rectangular region consisting of a slowly bleaching species (rate constant *k*_0_ = 0.01 s^−1^) contained a circular area with a faster bleaching species (rate constant *k*_1_ = 0.05 s^−1^) and a third elliptical region with a very fast bleaching species (rate constant *k*_2_ = 0.15 s^−1^). Thus, the three regions have the same initial intensity but differ in their bleaching kinetics. Accordingly, classical segmentation algorithms, such as Otsu, Triangle, Minimum or Li thresholding can only separate the rectangle from the background [34], but those algorithms fail in segmenting the circular and elliptical region when applied to the first frame of the bleach stack (Additional file 1: Fig. S1A and Additional file 2: Fig. S2–Additional file 4: Fig. S4).

Applying such algorithms to partially bleached frames (e.g., #10, 20 or 30) allows for segmenting the rectangular region but not the circular or elliptical region with only minor differences between the used thresholding methods (Additional file 1: Figs. S1B, C and Additional file 2: Fig. S2–Additional file 4: Fig. S4). When applying multi-Otsu thresholding to selected frames of the simulated bleach stack, all three regions could be segmented and separated from the background for frame #10, but not for frame #20 or frame #30, where the elliptical region could not be identified (Additional file 5: Fig. S5). Thus, without prior knowledge about which frame to analyze and the number of regions to be identified, segmentation of individual frames of a bleach stack is insufficient.

**Fig. 2:**
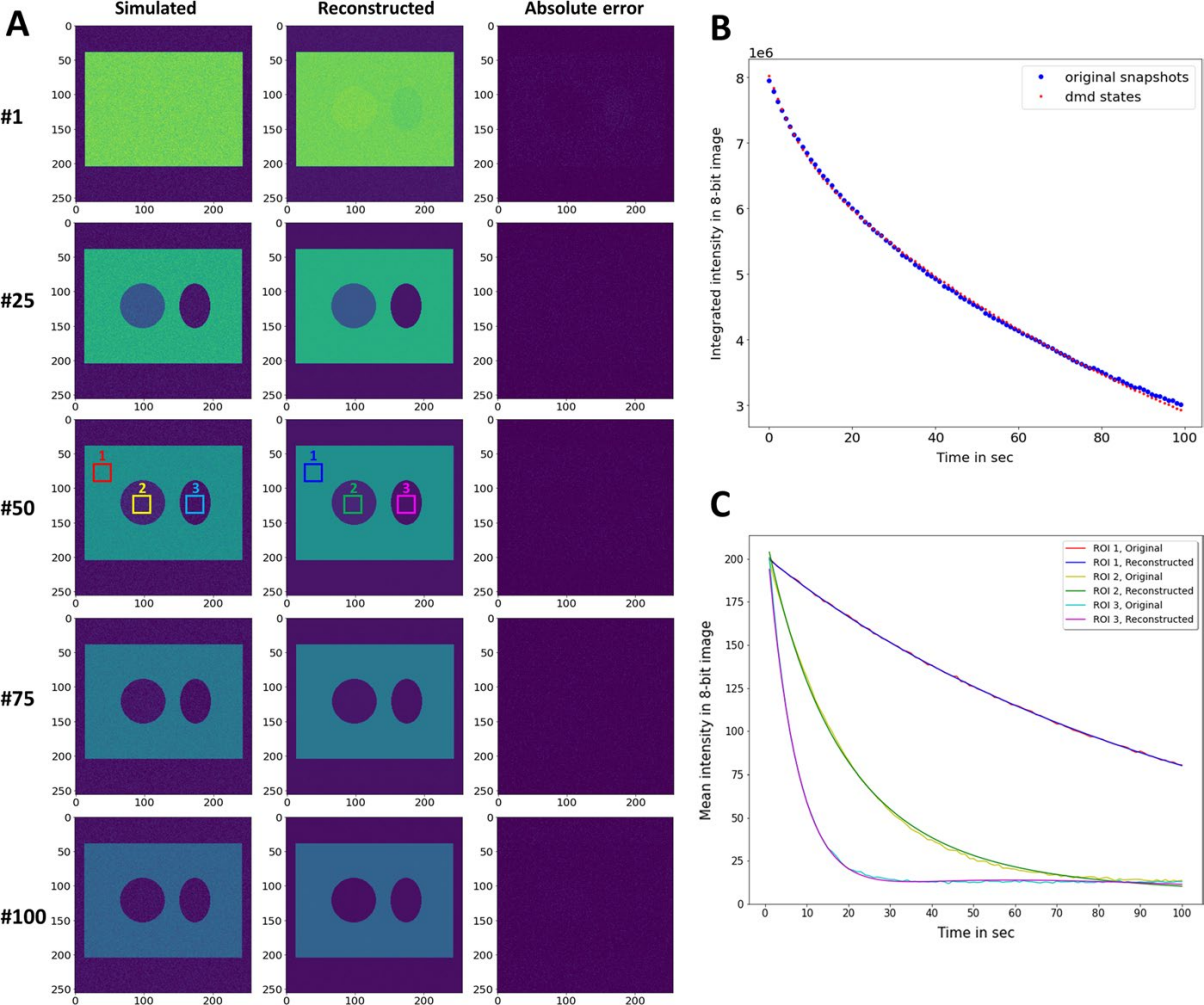
Comparison of simulated and reconstructed bleach stacks. **A**, selected frames (#1, #25, #50, #75 and #100) are plotted for the simulated bleach stack (left column) and the reconstructed image stack obtained from the DMD of the synthetic image series (middle column). Right column, absolute error between simulation and reconstruction. The intensity range is color-coded between 0 and 255 intensity units. One can see that DMD approximates the simulated images very well and is also efficient in removing image noise. **B**, integrated intensity of original (blue symbols) and reconstructed image stacks (red symbols). **C**, mean intensity in color-coded boxes (see #50 in A for location of regions of interest, ROI) for original (red, yellow and cyan lines) and reconstructed video stacks (blue, green and pink lines)

**Fig. 3:**
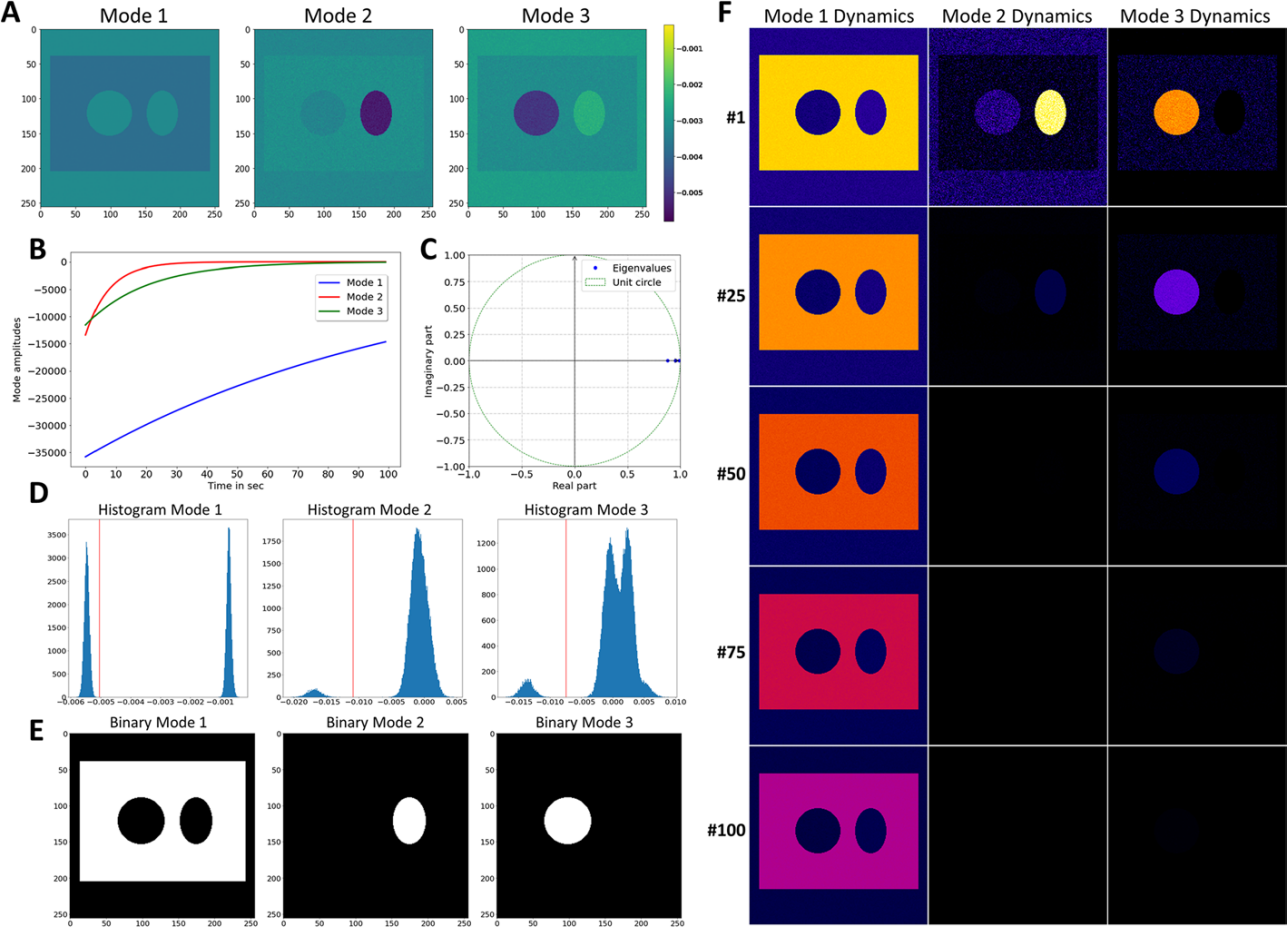
Dynamic mode decomposition enables accurate segmentation of synthetic bleach stacks. A rank-3 approximation of the full matrix A was employed to decompose the simulated bleaching kinetics. **A**, 2D map of dynamic modes, i.e., Mode 1 (*φ*_1_), Mode 2 (*φ*_2_) and Mode 3 (*φ*_3_). **B**, mode dynamics for each of the three modes and **C**, eigenvalues for each mode, ω_1_ to ω_3_, plotted on the unit circle. **D**, histogram of each dynamic mode (compare panel **A**) with threshold intensity value determined by the Minimum method indicated as red line. **E**, result of binary segmentation with white being foreground and black being background. While thresholding Mode 1 segments the slowly bleaching rectangular region, thresholding Mode 2 isolates the elliptical region with fast bleaching kinetics. Thresholding Mode 3 isolated the circular region with intermediate bleaching kinetics. **F**, reconstructed mode dynamics according to Eq. 11 for Mode 1 (left column), Mode 2 (middle column) and Mode 3 (right column)

It needs methods which account for the entire bleaching dynamics in an image stack simultaneously, such as DMD, for proper image segmentation and separation of several fluorophores in the same sample. DMD of this bleach stack was carried out using an automated threshold determination based on the highest singular values, which resulted in a rank-3 approximation of the full data matrix [35]. That means, only three dynamic modes are needed to capture the relevant dynamics in this system. As shown in Fig. 2, such a rank-3 approximation of the system matrix is sufficient to describe the image data adequately. Its dynamics is very well captured by the reconstruction with both, the integrated intensity of the whole stack and the mean intensity in selected regions, both being in good agreement with the original data. The rank-truncation in the DMD reconstruction of this simulated bleach stack has the additional benefit that most noise is removed, such that the different regions can be better discerned than in the simulated original data (Fig. 2A). Thus, DMD can be used to denoise the bleach stacks, which improves the ability to segment regions based on bleaching kinetics (Fig. 3). All three dynamic modes contain negative values and have negative mode amplitudes, such that their product according to Eq. 11 gives exponential decays from initially positive values (Fig. 3A, B). Accordingly, the more negative the pixel values in the 2D maps of dynamic modes are (blue in Fig. 3A), the higher is the contribution of this region to the mode decay. For Mode 1, this is the rectangular region (Fig. 3A), which has the slowest mode dynamics (Fig. 3B, blue line), in accordance with its slow bleaching kinetics (Fig. 2A, C). For Mode 2, most negative values are found in the elliptical region (Fig. 3A), which has the fastest mode dynamics (Fig. 3B, red line) and also the fastest bleaching kinetics (Fig. 2A, C). Mode 3 has the most negative values in the circular region (Fig. 3A), which has an intermediate mode dynamic (Fig. 3B, green line). This agrees with the intermediate bleaching rate constant defined for this region (Fig. 2A, C). Thus, DMD captures the individual bleaching dynamics of different image regions very well. There are three real eigenvalues, one for each mode (Fig. 3C). The eigenvalues are upon logarithmic scaling (Eq. 12) equal to ω_1_ = − 0.009 s, ω_2_ = − −0.128 s and ω_3_ = − 0.05 s for dynamics modes 1, 2 and 3, respectively. This is similar but not identical to the eigenvalues used in the simulations, i.e., to − *k*_0_ = − 0.01 s^−1^ for the rectangular region (≈ ω_1_),  − *k*_2_ = − 0.15 s^−1^(≈ ω_2_) for the elliptical region and − *k*_1_ = − 0.05 s^−1^ ((≈ ω_3_) for the circular area of the simulated bleach stack, respectively. Note that these eigenvalues are not expected to precisely resemble the rate constants used in the simulations, since DMD is not a model fitting technique. Instead, the dynamics at each pixel position is described as the sum of all three dynamic modes multiplied by the exponentials and mode weights (see Eq. 11). Image regions, which are not or only very little contributing to a given mode, have zero or very small entries in their 2D map of the corresponding dynamic mode (Fig. 3A). Based on this feature, the dynamic modes can be used to threshold bleach stacks, thereby segmenting the images into regions of different bleaching dynamics despite identical intensities in the first image. This is illustrated in Fig. 3D, E for the simulated bleach stack, in which the Minimum-based thresholding method was used to segment Mode 1 into background and rectangular region (without circle and ellipse) as foreground, Mode 2 into elliptical region and background and Mode 3 into circular region and background, respectively [34, 36].

Image thresholding was applied separately to Mode 1, 2 and 3 of the DMD of the synthetic bleach stack and compared to ground truth images for the rectangular region without circle and ellipse inscribed (Ground truth 1), the elliptical region (Ground truth 2) and the circular region (Ground truth 3). The Jaccard index, J, and the Dice score, D, are provided for different thresholding methods; compare to Fig. 3E.

Segmentation quality is very high as inferred from measuring the Jaccard index ($J(y,\widetilde{y})$) and Dice score ($D(y,\widetilde{y})$, Eqs. 13 and 14), which both provided values of 1.0, i.e., a perfect overlap [37]. A systematic analysis of all employed thresholding methods confirms this conclusion (see Table 1). For comparison, pixel-wise bleach rate fitting of the bleach stack using a mono-exponential decay function provides precise estimates of the rate constants and a comparable reconstruction quality as DMD (not shown) [8]. However, this method allows only for segmentation of one region by the Minimum method (i.e., either the rectangular region, when using the time constant image with $J\left(y,\widetilde{y}\right)=0.999, D\left(y,\widetilde{y}\right)=0.999$ or the elliptical region, when using the reciprocal, i.e., the rate constant image with $J\left(y,\widetilde{y}\right)=1.00, D\left(y,\widetilde{y}\right)=1.00$, Additional file 2: Fig. S2–Additional file 4: Fig. S4 and Additional file 6: Fig. S6). Similarly, other histogram-based segmentation methods fail in correctly identifying all three regions based on the time constant or rate constant maps provided by PixBleach (Additional file 2: Fig. S2–Additional file 4: Fig. S4 and Additional file 7: Fig. S7). When applying multi-Otsu thresholding [38], the circular and elliptical region could be separated based on the rate constant image in PixBleach [8], but the method failed to segment the rectangular region in parallel (Additional file 8: Fig. S8). In contrast, the sum of Mode 2 and 3 determined by DMD could be segmented into all four regions, though with lower accuracy then when thresholding the three different modes individually (compare Fig. 3 and Additional file 8: Fig. S8). In summary, DMD-based segmentation of image stacks based on differences in bleaching kinetics outcompetes classical thresholding methods and is at least on par with other bleaching-based approaches for image segmentation. DMD has the additional advantage that each dynamic mode can be separately visualized and analyzed. This is illustrated in Fig. 3F, which shows that multiplying each mode with the corresponding mode amplitudes and exponentials of eigenvalues according to Eq. 11 provides the time-evolution of each dynamic mode. One can clearly see that Mode 1 captures regions with the slowest dynamics (the rectangular area without inscribed circle and ellipse) and Mode 2 those with the fastest dynamics (the elliptical area). Mode 3 is comprised of pixels with intermediate decay dynamics (the circular area). This is in very good agreement with the segmentation results and demonstrates that the reconstructed dynamic modes correctly capture the bleaching dynamics in each individual image region. Thus, applying DMD to bleach stacks makes that additional image segmentation using e.g., thresholding methods is not strictly necessary anymore. This is a very important conclusion of this study, as it provides a new paradigm for isolating different fluorophores solely based on their characteristic bleaching kinetics, even if there is no difference in intensity.

**Table 1:** Quantification of segmentation accuracy of DMD for the three different image regions

Method	Ground truth 1 vs. mode 1	Ground truth 2 vs. mode 2	Ground truth 3 vs. mode 3
Triangle	J = 0.999	J = 0.996	J = 0.998
	D = 0.999	D = 0.998	D = 0.999
Minimum	J = 1.000	J = 1.000	J = 1.000
	D = 1.000	D = 1.000	D = 1.000
Isodata	J = 1.000	J = 1.000	J = 1.000
	D = 1.000	D = 1.000	D = 1.000
Li	J = 1.000	J = 1.000	J = 1.000
	D = 1.000	D = 1.000	D = 1.000
Mean	J = 1.000	J = 0.651	J = 0.606
	D = 1.000	D = 0.789	D = 0.755
Otsu	J = 1.000	J = 1.000	J = 1.000
	D = 1.000	D = 1.000	D = 1.000
Yen	J = 0.995	J = 0.988	J = 0.988
	D = 0.997	D = 0.994	D = 0.994

### DMD of experimental image stacks of photobleaching in C. elegans

**Fig. 4:**
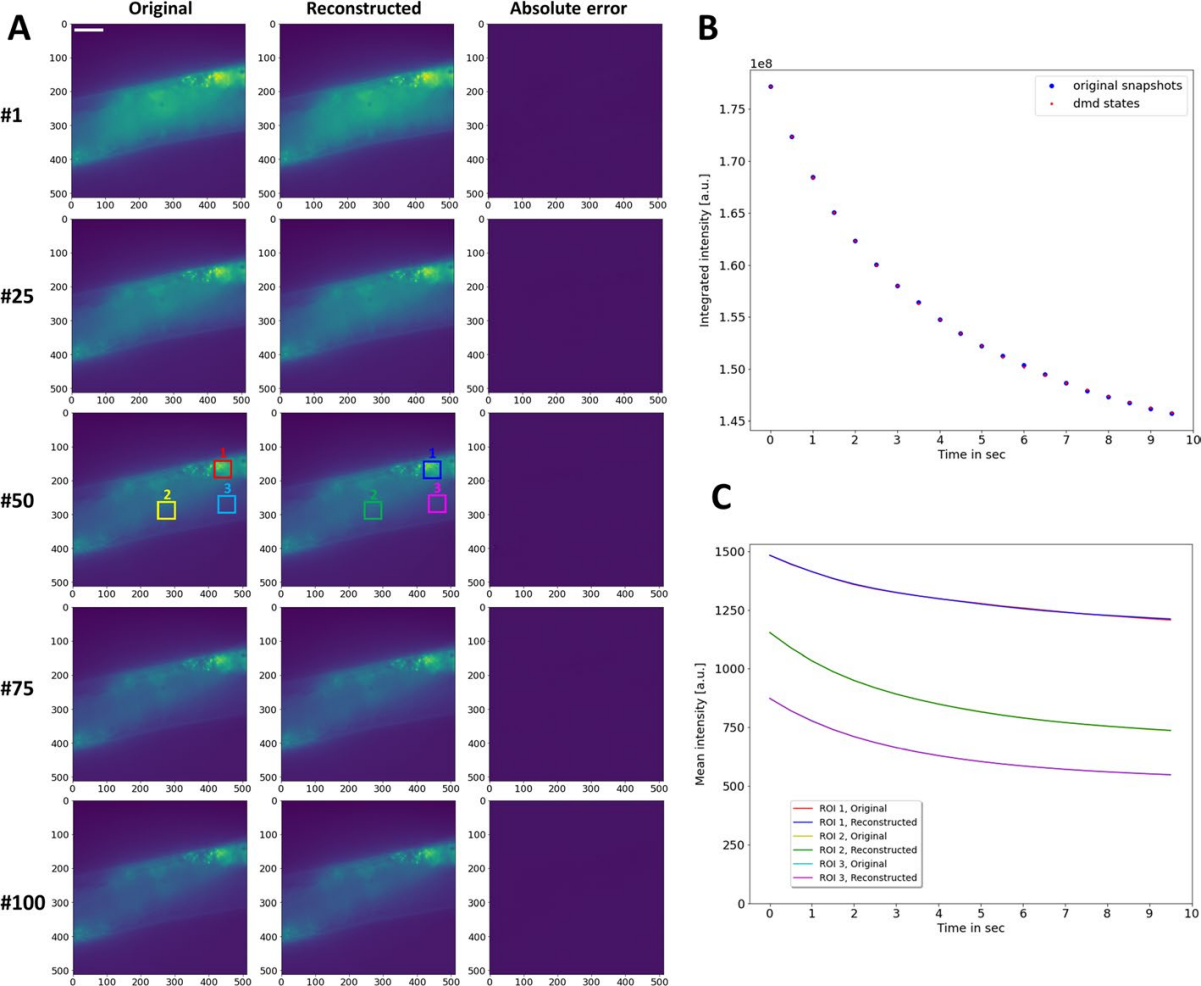
Comparison of experimental and reconstructed bleach stacks of DHE in *C. elegans*. **A**, **B** montage of selected frames (i.e., frame (#1, #25, #50, #75 and #100) of the experimental fluorescence stack of DHE labeled *C. elegans* (left column) and of the reconstructed image stack obtained from the DMD of rank 5 (right column). Right column, absolute error between original stack and DMD reconstruction. The intensity range is identically scaled in 16-bit format. **B**, integrated intensity of original (blue symbols) and reconstructed image stacks (red symbols). **C**, mean intensity in color-coded boxes (see #50 in A for location of regions of interest, ROI) for original (red, yellow and cyan lines) and reconstructed video stacks (blue, green and pink lines). Note that intensities of the DMD reconstruction perfectly coincide with the intensities of the original stack; therefore, only the line colors of the reconstruction are visible. Bar, 20 µm. See text for further details

**Fig. 5:**
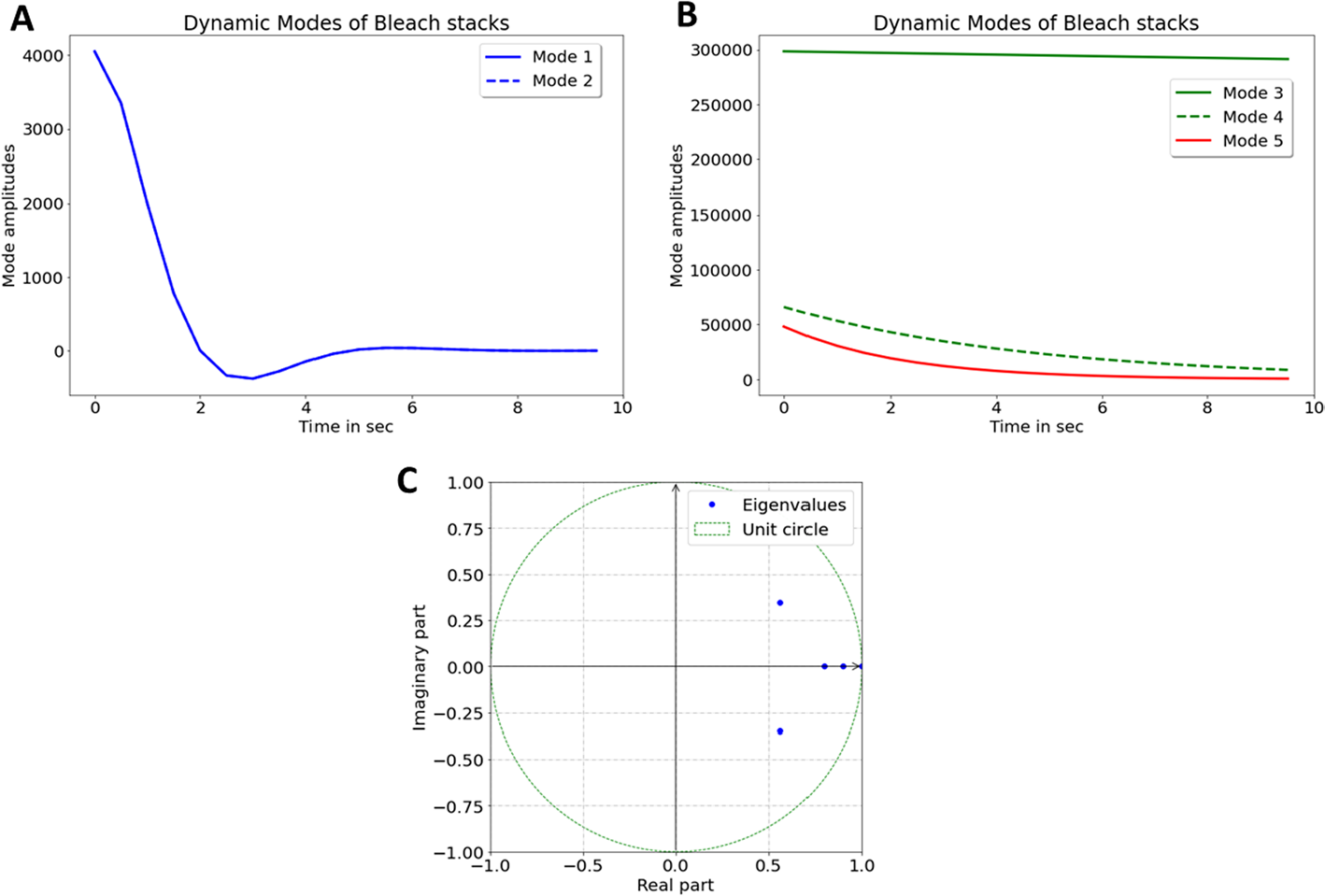
Dynamic mode amplitudes and eigenvalues of fluorescence images of DHE-labeled nematodes. **A**, **B**, real part of mode amplitudes of a rank-5 DMD of the experimental bleach stacks of DHE labeled *C. elegans* with two oscillatory modes (Mode 1 and 2, A) and three exponentially decaying amplitudes (Mode 3–5, B). **C**, eigenvalues λ_1_ to λ_5_ plotted on the unit circle. The first two eigenvalues have non-zero imaginary part (see also the corresponding oscillatory amplitudes in A). Eigenvalues 3–5 are real and smaller than one, describing decaying intensities in the bleach stacks

**Fig. 6:**
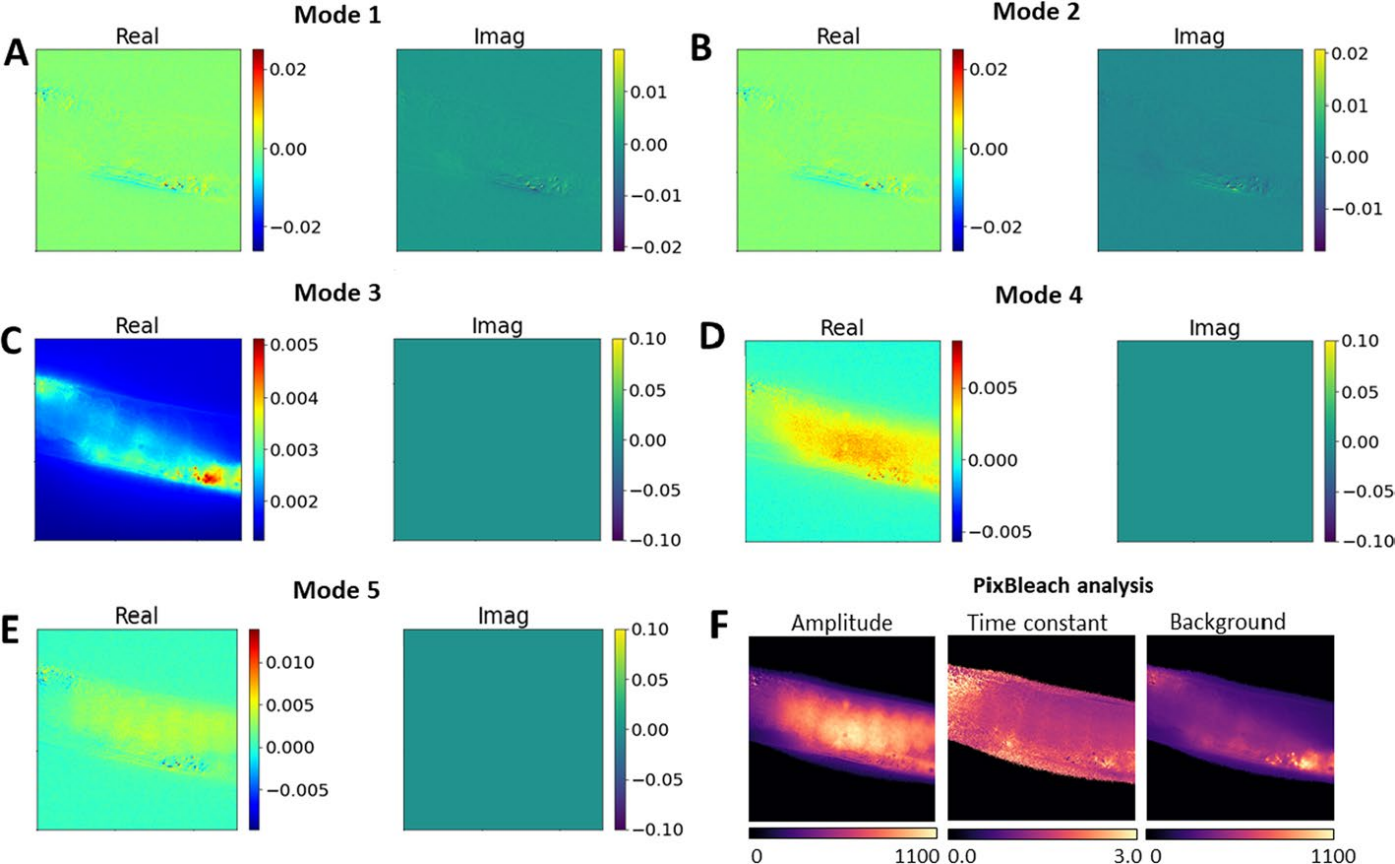
Dynamic modes of C.elegans bleach stacks and comparison with pixel-wise fitting. **A**–**E**, mode weights for DMD modes 1–5. The real part of mode weights is shown in left panels (‘Real’), while the imaginary parts are shown in right panels (‘Imag’). **F**, bleach rate fitting using a stretched exponential function with bleaching amplitudes (right panel), time constant map (middle panel) and background fluorescence (right panel). The amplitude image in **F** shows the distribution of the rapidly bleaching DHE, while the background image resembles most of the autofluorescence

To assess, whether DMD can be used to segment real experimental data, *C. elegans* nematodes labeled with the intrinsically fluorescent sterol DHE were repeatedly imaged. Our earlier studies showed that DHE’s fluorescence emission strongly overlaps with autofluorescence of nematodes in the ultraviolet region of the spectrum, but also that DHE bleaches much faster than autofluorescence [8, 9]. To decompose the differential bleaching of probe and autofluorescence, image stacks were analyzed by DMD using a rank-5 approximation of the full data matrix. Strikingly, the reconstructed image stack containing the information from all five dynamic modes perfectly matches the original data, and the decreasing integrated intensity of both image stacks coincides closely (Fig. 4A, B). Also, the bleaching kinetics in individual regions perfectly coincides for the original image stack and the DMD reconstruction (i.e., intensity decay profiles differ by less than 0.2% for all intensities and therefore overlap in the curves, Fig. 4C). DMD of this fluorescence image series resulted in two complex eigenvalues, which describe two degenerated modes with oscillating dynamics (Fig. 5A, ‘Mode 1 and 2’ and C). In addition, there are three real eigenvalues smaller than one, corresponding to decaying dynamic modes, which describe the bleaching kinetics of DHE and autofluorescence respectively (Fig. 5B ‘Mode 3–5’ and C). Inspection of the mode weights confirms this interpretation (Fig. 6); Mode 1 and 2 contain non-zero imaginary parts, which account for the slight lateral displacement of the nematodes during imaging (Fig. 6A, B). Such movement cannot be entirely prevented despite anesthetizing the animals before imaging, and it can impact the fit quality in pixel-wise bleach rate fitting [8]. In contrast, in DMD one can account for some movement of subcellular structures without compromising the bleaching analysis. Mode 3–5 describe the decaying intensity, as inferred from the mode weights which all have only real entries (Fig. 6C–E). These modes have real eigenvalues, smaller than one and decaying mode amplitudes (Fig. 5B, C). Mode 3 described the slowly decaying autofluorescence, while Mode 4 and 5 describe bleaching of the fluorescent sterol DHE. This interpretation is supported by comparing the outcome of DMD with bleach-rate fitting, which shows that significant bleaching takes place in the region of the oocytes and intestinal cells, where the DHE resides (see ‘Amplitude’ image in Fig. 6F), closely resembling the mode weight images for Mode 4 and 5 (Fig. 6D, E).

We and others showed previously that oocytes are particularly sterol-rich cells in *C. elegans*, since the ingested sterols are essential for steroid hormone production to control developmental transitions [7, 8, 39, 40]. Pixel-wise bleach rate fitting identifies autofluorescence as non-bleaching, i.e., constant background term (Fig. 6F). That background map is very similar to the real peart of the weight image of Mode 3 of the DMD (compare Fig. 6C, left panel and F, right panel). Having decomposed the entire dynamics in the bleach stacks of nematodes, DMD allows for separate inspection and analysis of each dynamic mode. This makes it possible to separate autofluorescence from DHE intensity and thereby to segment the images into different fluorescence contributions. From the DMD one can calculate image stacks representing the individual dynamic modes as a decomposition of the entire bleaching dynamics in the original image stacks (see Eqs. 11 and 12).

**Fig. 7:**
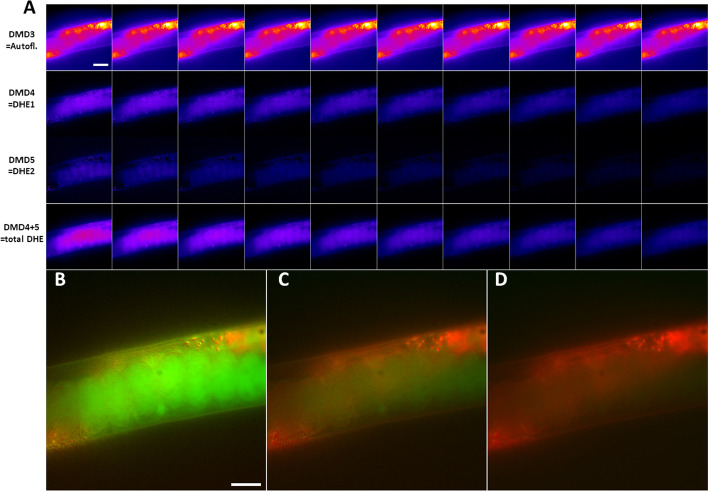
DMD of *C. elegans* bleach stacks allows for discrimination of autofluorescence from DHE probe intensity. **A**, montage of individual dynamic modes shown as every second image of the corresponding image stacks. Dynamic mode 3 (‘DMD3’) resembles cellular autofluorescence of nematodes (upper row in A), while dynamic mode 4 and 5 (‘DMD4’ and ‘DMD5’) constitute DHE fluorescence (two middle rows in A). The sum of mode 4 and 5 shows the total DHE fluorescence (lower row in A). **B**–**D**, color overlay of mode decomposition with mode 3 resembling autofluorescence in red and sum of mode 4 and 5 representing DHE fluorescence in green. **B**, first frame, **C**, 10th frame and **D**, 20th frame of this color representation of the DMD, showing the rapid bleaching of DHE fluorescence (green) compared to autofluorescence (red). Bar, 20 µm

From that, one can clearly infer cellular autofluorescence from Mode 3 and more rapidly decaying DHE fluorescence from Mode 4 and 5, respectively (Fig. 7). Clearly, the sum of Mode 4 and 5 resembles the total DHE fluorescence, which bleaches much faster than cellular autofluorescence. Accordingly, DMD allows for segmentation of image structures based on their dynamics, which makes it possible that one pixel contains information from both dynamic structures, just to different extent, as visualized in a color overlay of the decomposed bleaching dynamics (Fig. 7B–D). This is not straightforward to implement in pixel-based fitting, where pixel overlap of two regions can only be accounted for by bi-exponential fitting, which often fails, when the signal-to-noise ratio in image regions gets to small [8]. In addition, DMD but not pixel-wise fitting provides the bleaching dynamics of each fluorescent entity as separate image stacks.

To further explore the potential of DMD for discriminating probe fluorescence from autofluorescence control experiments were carried out using *C. elegans* mutants, which lack gut granules and have therefore reduced autofluorescence [41]. DMD of these stacks allows for a very good reconstruction quality similar to wild-type cells (Additional file 9: Fig. S9A). The remaining autofluorescence in these animals was estimated as the slowest bleaching dynamic mode (Mode 1 with eigenvalue ω_1_ = − 1∙10^–5^ s; Additional file 9: Fig. S9B and C), as in control animals (compare with Figs. 4, 5, 6 and 7). Both, DHE fluorescence and remaining autofluorescence overlapped in the intestine of those animals, as inferred from co-localization with eGFP-tagged rab5, a marker for early endosomes, abundantly expressed in the intestine of *C. elegans* (Additional file 9: Fig. S9D) [42]. Co-localization was particularly pronounced in the basolateral domain of intestinal epithelial cells, a region, where early recycling endosomes reside in *C. elegans* [43]. Since recycling endosomes are known to be sterol-rich in epithelial cells of various organisms, this is a further validation of our method [44, 45]. DHE was additionally detected in the pharynx region and the lumen of the intestine, as expected, since the nematodes acquire the sterols by feeding [7, 8].

### DMD of experimental image stacks of mammalian cells labeled with two green-fluorescent probes

To further explore the potential of the method, DMD was used to discriminate two fluorescence tagged molecules, which are spectrally indistinguishable, but show different bleaching kinetics in living cells. BHK cells were labeled with two green emitting fluorescence probes; the iron-transporting protein transferrin tagged with an Alexa488-dye, which is very photostable (Additional file 10: Fig. S10) and the membrane probe C6-NBD-SM, which bleaches much faster (Additional file 11: Fig. S11). Alexa488-Tf will bind to the transferrin receptor and become internalized by clathrin-mediated endocytosis followed by recycling from early sorting and recycling endosomes [46]. The latter is also called the endocytic recycling compartment (ERC) and appears as perinuclear enrichment of small vesicles in Chinese hamster ovarian (CHO) and BHK cells [47, 48]. C6-NBD-SM is a fluorescent sphingolipid probe, which has been shown to be targeted to the ERC and recycled from the cell with very similar kinetics as Tf, which is why this fluorescent lipid probe is often seen as ‘bulk membrane recycling marker’ [47, 49–51]. Thus, both probes accumulate in the perinuclear ERC, but to different extent. While the majority of C6-NBD-SM remains in the plasma membrane (PM), almost the entire pool of Alexa488-Tf will accumulate in the ERC in this experiment. By loading C6-NBD-SM onto albumin, the lipid probe can be rapidly inserted into the PM, followed by its endocytosis and trafficking through the endocytic recycling pathway together with fluorescent Tf [51]. Such co-trafficking is normally assessed in two-color fluorescence imaging experiments, in which the emission color of the fluorescent probes is separated using suitable filter combinations [49–51].

**Fig. 8:**
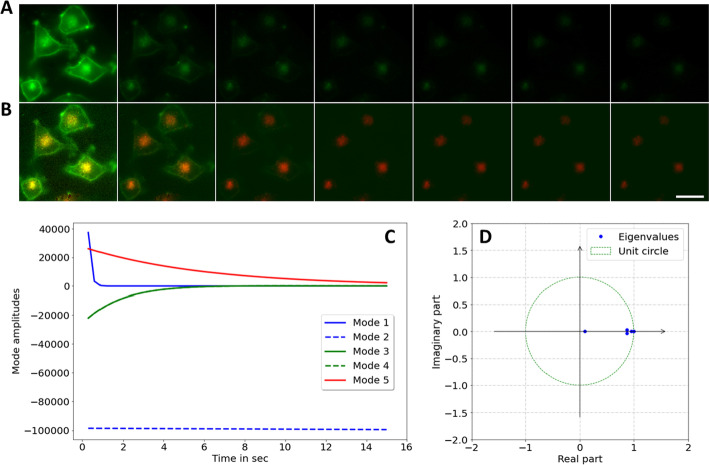
DMD of bleach stacks of BHK cells labeled with two green probes, Alexa488-Tf and C6-NBD-SM. BHK cells were labeled with 4 µM C6-NBD-SM and with 20 µg/ml Alexa488-Tf, both emitting in green, as described in Materials and Methods. **A**, montage of selected frames (every 5th frame) of such double labeled cells. **B**, reconstruction of DMD of the bleach stack in A with the sum of dynamic modes 1, 3 and 4 shown in green (resembling the fast-bleaching C6-NBD-SM) and sum of dynamic mode 2 and 5 (resembling the slowly bleaching Alexa488-Tf) shown in red. Bar, 20 µm. **C**, mode amplitudes and **D**, eigenvalues of the DMD plotted on the unit circle

Using different colors for two endocytic markers limits the number of additional channels to be available for other probes to two on most wide field and confocal microscopy systems (e.g., there is typically an additional blue filter set for DAPI and an infrared filter cube for another organelle marker). By decomposing the different bleaching kinetics of the two green emitting endocytic probes, Alexa488-Tf and C6-NBD-SM using DMD, their intracellular distribution can be determined using only one filter set (Fig. 8). As shown in Fig. 8A, the green fluorescence bleaches rapidly in the PM, where a major portion of C6-NBD-SM resides but much slower in the ERC, where the majority of the more photostable Alexa488-Tf is located. This notion is sustained by DMD of image stacks of cells labeled with only Alexa488-Tf (Additional file 10: Fig. S10) or only with C6-NBD-SM (Additional file 11: Fig. S11). The heterogeneous bleaching dynamics of doubly labeled cells can be decomposed by DMD into five dynamic modes, three of which show fast decay (Mode 1, 3 and 4; Fig. 8C). Mode 2 is almost constant and has an eigenvalue, λ_2_, close to one (corresponding to ω_2_ ≈ 0.00; Eq. 12, Fig. 8D), while Mode 5 decays slowly (dashed blue and straight red line in Fig. 8C). From the corresponding mode weight maps, one sees that Mode 1, 3 and 4 have intensity in the PM and in the ERC, while Mode 2 and 5 have non-zero intensities almost exclusively in the perinuclear area (Additional file 12: Fig. S12). Based on these observations, the sum of Mode 1, 3 and 4 are assigned to the lipid marker C6-NBD-SM, while the sum of Mode 2 and 5 are assigned to Alexa488-Tf. The intracellular distribution of C6-NBD-SM and Alexa488-Tf overlaps in the perinuclear ERC but only very little the PM (see color overlay in Fig. 8B with C6-NBD-SM in green and Alexa488-Tf pseudo-colored in red). Clearly, while C6-NBD-SM bleaches rapidly (Fig. 8B, green), Alexa488-Tf does bleach as well but much more slowly (Fig. 8B, red). Both conclusions are confirmed from stacks of single-labeled cells (Additional file 10: Fig. S10 and Additional file 11: Fig. S11). In pixel-wise bleach rate fitting one cannot separate the rapidly bleach lipid probe from the slowly bleaching Alexa488-Tf, which instead would be assigned to the background term (Additional file 12: Fig. S12F). These results demonstrate the potential of DMD in decomposing photobleaching dynamics for efficient separation of different fluorophores in live-cell microscopy.

## Discussion

In this study, it is shown that image segmentation and separation of spectrally indistinguishable fluorophores in live-cell microscopy can be achieved by DMD of their photobleaching kinetics. This is demonstrated first on synthetic image stacks with simulated photobleaching and thereafter on two different experimental data sets. In all cases, DMD can decompose the photobleaching kinetics properly, allowing for clear separation of probe from autofluorescence or of different fluorescent dyes in the same sample. One can therefore envision that DMD of probe photobleaching can be combined with spectral unmixing, thereby increasing the number of probes which can be detected in parallel [1, 19]. Additionally, DMD can compensate for noise and small movement artefacts, making it a powerful computational tool for analyzing photobleaching in live-cell imaging experiments in the future. There is a strong interest in developing novel fluorescent probes for biomedical imaging applications. Here, a concern is often that prolonged fluorescence imaging of dynamic processes in living cells can lead to photo destruction of fluorophores [52]. Therefore, it is important to determine photobleaching characteristics of fluorescent dyes under various conditions, either for optimizing probe design or for optimizing imaging conditions [14, 20, 53, 54]. For example, sensitive single molecule imaging and super-resolution microscopy critically depends on development of bright and photostable fluorophores and here, DMD of dye photobleaching in cells can be very useful for designing improved organic fluorophores [55]. DMD will also be useful in discriminating different fluorophores based on their characteristic bleaching propensities, for multi-color super-resolution microscopy [56]. Similarly, optimizing photosynthetic antenna systems for light harvesting applications in photovoltaics demands photostable pigment structures, and analysis of their photo destruction using imaging-based DMD can be combined with other approaches such as lifetime and absorption measurements as well as electron paramagnetic resonance spectroscopy in the future [57].

Furthermore, photobleaching kinetics also report about environmental impacts on a fluorophore, since in many cases, photobleaching kinetics is inversely related to the fluorescence lifetime of a fluorophore. For example, if a nearby acceptor molecule can receive the energy of an excited fluorophore due to Förster resonance energy transfer (FRET), its excited-state lifetime gets reduced and accordingly its bleaching propensity lowered, since photobleaching can only take place from an excited state. Therefore, photobleaching kinetics of the donor molecule can report about FRET efficiencies, similar as lifetime imaging [22, 58, 59]. Under conditions, where lifetime imaging is not feasible or at least very difficult, e.g., for weakly emitting UV probes, analysis of donor photobleaching kinetics is a very useful approach and here DMD is a good tool for their analysis. The same applies to quenching studies, in which a dynamic quencher is used to determine the accessibility of a fluorescent probe, for example when analyzing the permeability of the fungal cell wall [60], or the transbilayer distribution of a membrane probe between the two PM leaflets [61–64]. Dynamic quenching shortens the fluorescence lifetime and thereby slows the photobleaching of the quenched dye [10, 13, 65], which can be detected by DMD of its photobleaching kinetics. The extent of photobleaching is often directly proportional to the occupation of the triplet state of a fluorophore, from which reaction with singlet oxygen and thereby photooxidation can take place. This concept is used in photodynamic therapy, where light-induced production of reactive oxygen species is used to kill tumor cells selectively [66–68]. Here, control of the photobleaching process is essential, and DMD of photobleaching kinetics can become a useful tool in its analysis.

## Conclusions

A new computational method is presented to analyze photobleaching kinetics of fluorescent entities in microscopy images of living cells in a purely data-driven manner. It is shown that the decomposition of photobleaching kinetics into dynamic contributions allows for image segmentation, image denoising and discrimination of different fluorescent probes and autofluorescence on a pixel-by-pixel basis. This novel approach can be combined with spectral unmixing and FRET studies, for example as part of large-scale image-based screens to assess organelle and marker distribution in multi-color experiments.

## Materials and methods

### Materials

N-[6-[(7-nitro-2-1,3-benzoxadia-zol-4-yl)amino]-dodecanoyl]-sphingosine-1-phosphocholine (C6-NBD-SM) was obtained from Avanti Polar Lipids (Alabaster, AL). DHE was purchased from SIGMA Chemical (St. Louis, MA). Buffer medium contained 150 mM NaCl, 5 mM KCl, 1 mM CaCl2, 1 mM MgCl2, 5mM glucose and 20m MHEPES (pH 7.4). Alexa488-protein labeling kit was purchased from Molecular Probes (ThermoFisher). Transferrin was iron loaded as described previously [69]. The succinimidyl ester of Alexa-588 was conjugated to the iron-loaded transferrin to get Alexa488-Tf following the manufacturer’s instructions. C6-NBD-SM was loaded onto fatty-acid free bovine serum albumin (BSA) following our previously published procedure [70]. BHKasc cells were kindly provided by Dr. Kirsten Sandvig, Cancer Center, Norwegian Radiation Hospital, University of Oslo, Norway. Gut granule loss (glo-) mutant strains of C.elegans were kindly provided by Dr. Greg Hermann, Department of Biology, Lewis and Clark College, Portland, OR, USA.

### Labeling and imaging of nematodes

Wild-type *C. elegans* or glo-mutant strains or were cultured, labeled with DHE and imaged on a UV-sensitive wide field microscope, exactly as described previously [8]. Images of nemtatodes were acquired in the UV channel (335 nm (20 nm bandpass) excitation filter, 365 nm dichromatic mirror and 405 nm (40 nm bandpass) emission filter) with an acquisition time of 500 ms and no pause between acquisitions.

### Culture, labeling and imaging of Baby hamster kidney (BHK) cells

BHKasc cells were grown in DMEM supplemented with 7.5% heat-inactivated FCS, 2 mM l-glutamine, 100 units/ml penicillin, 100 µg/ml streptomycin, 0.2 mg/ml geneticin, and 2 µg/ml tetracycline [71]. Three days prior to the experiments, the cells were seeded on microscope slide dishes and kept in the same medium until the experiments. Cells were labeled with 4 µM C6-NBD-SM for 5 min at 37 °C and washed three times with buffer medium. Subsequently, cells were labeled with 20 µg/ml Alexa488-Tf for 30 min at 37 °C and washed three times with buffer medium. In separate control experiments, cells were either labeled just with C6-NBD-SM, washed, and chased for 30 min or just with Alexa488-Tf for 30 min at 37 °C and washed three times with buffer medium before imaging on a widefield microscope, as described for imaging of nematodes, just with standard fluorescein filter sets ([470-nm, (20-nm bandpass) excitation filter, 510-nm longpass dichromatic filter and 537-nm (23-nm bandpass) emission filter]).

### Image simulation, segmentation and data analysis

To validate the procedure synthetic bleach stacks with known bleaching characteristics were generated using the Macro language of ImageJ (https://imagej.nih.gov/ij/), as described previously [8, 72]. Specifically, an 8-bit image stack with a background of random intensities with mean intensity equal to 10 was generated in which a rectangular region of mean intensity 190 contained one circles and one ellipse with mean intensity of 190 each. The intensity decayed monoexponentially in the rectangular region excluding circle and ellipse with rate constant *k*_0_ = 0.01 s^−1^, in the circular area with rate constant *k*_1_ = 0.05 s^−1^ and in the elliptical region with rate constant *k*_2_ = 0.15 s^−1^. DMD and accompanying analysis was carried out in Python using Jupyter notebooks (https://jupyter.org/) and PyDMD, a python library for DMD calculations [73]. In brief, upon SVD of the image data matrix with either a pre-defined rank or matrix-specific optimal rank, the DMD modes are calculated. Eigenvalues determined for a rank-*r* decomposition of the system matrix *A*, λ_j_, for *j* = 1,…, *r*, are logarithmically scaled and divided by the interval time (i.e. the acquisition time for bleach stacks, Δt):

12 $$\omega_{j} = log\left( {\lambda_{j} } \right)/{\Delta }t$$

Using the calculated dynamic modes, *φ*_j_, and their amplitudes, *b*_j_, the time evolution of each dynamic mode is calculated according to Eq. 11, above. For pixel-wise bleach rate fitting a plugin to ImageJ, PixBleach, was used [8, 74]. Both methods were compared by calculating the root mean square error (RMSE) between data and model. Image segmentation was carried out using a variety of classical region-based thresholding methods (i.e., Otsu, Isodata, Li, Yen, Mean, Minimum and Triangle) implemented in skimage (http://scikit-image.org) using the skimage.filters.try_all_threshold method [34, 75]. While the Otsu method attempts to minimize the weighted sum of within-class variances of foreground and background pixels [34, 36], Isodata determines the threshold as the average intensity of two identified clusters based on the image histogram [76]. Li and Yen are entropy-based thresholding methods; Li and Lee (1993) minimize the cross-entropy between original and binarized image [77], while Yen et al. define and maximize the entropic correlation of foreground and background pixels [78]. Mean, Minimum and Triangle similarly employ the shape and moments of histograms for image thresholding [34, 79, 80]. All of the above thresholding methods were also applied individually, and the segmentation quality was assessed using the Jaccard index, *J*, defined as:

13 $$J\left( {A,B} \right) = \frac{{\left\lceil {A \cap B} \right\rceil }}{{\left\lceil {A \cup B} \right\rceil }}$$

Here, *A* is the binary image generated by a given thresholding method (i.e., the predicted segmentation) and *B* is the ground truth binary image (i.e., the true segmentation), both considered as sets of pixels. The numerator in Eq. 13 is the number of observations in both sets, while the denominator is the number of observations in either set [37]. In addition, the Dice score, *D*, was calculated between segmented and ground truth image according to:

14 $$D\left( {A,B} \right) = \frac{{2 \cdot \left\lceil {A \cap B} \right\rceil }}{\left\lceil A \right\rceil + \left\lceil B \right\rceil }$$

It also quantifies the pixel-wise degree of similarity between the predicted and ground truth segmentation. Both, *J* and *D*, can take values between 0 (no overlap) and 1 (perfect overlap) [37]. Addtional files 13, 14, 15, 16, 17, 18 contain simulated and experimental bleach stacks, on which the analysis, desribed here, was based.

## Supplementary Information

**Additional file 1: Fig. S1.** Comparison of image thresholding methods for segmentation of synthetic bleach stacks. Several standard thresholding methods, (i.e., Isodata, Li, Mean, Minimum, Otsu, Triangle and Yen method [34]) were assessed in their ability to correctly segment the three image regions of the synthetic bleach stacks. Based on the first image of the stack, all methods could segment the rectangular region from the background but could not dissect the circular and elliptical region (**A**). Based on the 10th frame of the bleach stack, Isodata, Li, Mean, Minimum and Otsu could segment the rectangular region without the elliptical region, but only the Triangle and Yen method could additionally separate the circular region (**B**). Similar results were found for the 20th and 30th image frame (**C** and** D** and Fig. S2).

**Additional file 2: Fig. S2.** Jaccard index and Dice score for image segmentation of rectangular region from the synthetic bleach stack using standard thresholding methods. **A**, the rectangular region without enclosed circular and elliptical region as ground truth image (white is foreground, black is background).** B**, Jaccard index, upper rows, and Dice score, lower rows, were calculated for the indicated thresholding methods applied to the 1st, 10th, 20th and 30th frame of the bleach stack as well as for the rate constant and time constant images derived from pixel-wise bleaching analysis in PixBleach relative to the ground truth image.

**Additional file 3: Fig. S3.** Jaccard index and Dice score for image segmentation of elliptical region from the synthetic bleach stack using standard thresholding methods.** A**, the elliptical region as ground truth image (white is foreground, black is background).** B**, Jaccard index, upper rows, and Dice score, lower rows, were calculated for the indicated thresholding methods applied to the 1st, 10th, 20th and 30th frame of the bleach stack as well as for the rate constant and time constant images derived from pixel-wise bleaching analysis in PixBleach relative to the ground truth image.

**Additional file 4: Fig. S4.** Jaccard index and Dice score for image segmentation of circular region from the synthetic bleach stack using standard thresholding methods.** A**, the circular region as ground truth image (white is foreground, black is background).** B**, Jaccard index, upper rows, and Dice score, lower rows, were calculated for the indicated thresholding methods applied to the 1st, 10th, 20th and 30th frame of the bleach stack as well as for the rate constant and time constant images derived from pixel-wise bleaching analysis in PixBleach relative to the ground truth image.

**Additional file 5: Fig. S5.** Multi-Otsu thresholding of synthetic bleach stack shows varying results depending on which frame is analyzed. Multi-Otsu thresholding was applied to the 1st, 10th, 20th and 30th frame of the bleach stack [38]. For the 1st frame (**A**), only two regions could be distinguished (brown is foreground, dark blue is background). For the 10th frame (**B**), four regions could be distinguished (brown, yellow and light blue are foreground of the rectangular, circular and elliptical region, respectively; dark blue is background). For the 20th frame (**C**) and 30th (**D**), three regions could be distinguished (brown and light green are foreground of the rectangular and circular region, respectively; dark blue is background). Left panels are original images of the synthetic stack, middle panels are histograms with identified threshold indicated in red, right panels are thresholding results with color-labeled regions.

**Additional file 6: Fig. S6.** Minimum-based thresholding of time and rate constant maps derived from pixel-wise fitting of exponential decay functions to synthetic bleach stack. Pixel-wise bleach rate fitting was applied to the synthetic bleach stack as described in Materials and methods [8]. The resulting time constant image (**A** and** B**) and rate constant image (**C** and** D**) were segmented using the Minimum threshold method. Left panel in** A** and** C** shows the time and rate constant maps, respectively, and right panels show the corresponding histograms. Left panels in** B** and** D** show segmentation results with foreground in white and background in black. Right panels in** B** and** D** show histograms with identified threshold indicated in red.

**Additional file 7: Fig. S7.** Comparison of image thresholding methods for segmentation of time and rate constant maps derived from pixel-wise fitting of exponential decay functions to synthetic bleach stack. Several standard thresholding methods, (i.e., Isodata, Li, Mean, Minimum, Otsu, Triangle and Yen method [34]) were assessed in their ability to correctly segment the three image regions of the time (**A**) and rate constant maps (**B**) derived from pixel-wise fitting of exponential decay functions to the synthetic bleach stack.

**Additional file 8: Fig. S8.** Multi-Otsu thresholding of DMD and pixel-wise bleach rate fitting outputs of synthetic bleach stack. Multi-Otsu thresholding was applied to Mode 3 of the DMD (**A**), to the sum of Mode 1 and 3 (**B**), to the sum of Mode 2 and 3 (**C**) and to the rate constant map derived from pixel-wise fitting of exponential decay functions to the synthetic bleach stack (**D**) [38]. Left panels are the original analyzed images, middle panels are histograms with identified threshold indicated in red, right panels are thresholding results with color-labeled regions. The number of identified regions for each image equals the number of red lines plus one (i.e., three regions in** A** and** B** and four regions in** C** and** D**).

**Additional file 9: Fig. S9.** Dynamic mode decomposition of fluorescence images of DHE-labeled glo-mutant nematodes with reduced autofluorescence.** A**, montage of selected images of original (upper row) and reconstructed (lower row) image sequence shown as every fifth image of the corresponding image stack. The lowest panel shows the absolute error between original and reconstructed image sequence with same intensity scaling. First five eigenvalues of space-time matrix of experimental image sequence (**B**) and corresponding dynamic modes (**C**) determined by DMD. Sum of dynamic mode 1, 3, 4 and 5 resemble total DHE fluorescence (upper left panel in green in** D**). Mode 2 resembles cellular autofluorescence of nematodes (upper right panel in red in** D**). These worms also express eGFP-rab5 as marker for early and recycling endosomes in their intestine (lower left panel in blue in** D**). Some of the DHE and autofluorescence signal overlap with eGFP-rab5 in the intestine (lower right panel in** D**).** E**, color overlay of mode decomposition with dynamic mode 2 resembling autofluorescence in red and sum of mode 1, 3, 4 and 5 representing DHE fluorescence in green. Bar, 20 μm.

**Additional file 10: Fig. S10.** Dynamic mode decomposition of image stacks containing Alexa488-Tf labeled cells. BHK cells were labeled with 20 μg/ml Alexa488-Tf for 30 min, washed with buffer medium and imaged on a wide field fluorescence microscope.** A**, selected frames of an image stack acquired with 0.3 sec acquisition time and without pause. Images are identically scaled; bar 10 μm.** B**,** C**, DMD of this image stack using a rank-5 approximation to the full transfer matrix.** B**, mode weights and** C**, mode amplitudes as function of time.

**Additional file 11: Fig. S11.** Dynamic mode decomposition of image stacks containing C6-NBD-SM labeled cells. BHK cells were labeled with 4 μM C6-NBD-SM for 30 min, washed with buffer medium and imaged on a wide field fluorescence microscope. A, selected frames of an image stack acquired with 0.3 sec acquisition time and without pause. Images are identically scaled; bar 10 μm.** B**,** C**, DMD of this image stack using a rank-5 approximation to the full transfer matrix.** B**, mode weights and** C**, mode amplitudes as function of time.

**Additional file 12: Fig. S12.** Mode weights for DMD of image stacks of BHK cells labeled with C6-NBD-SM and Alexa488-Tf. BHK cells were labeled with 4 μM C6-NBD-SM and with 20 μg/ml Alexa488-Tf, both emitting in green, as described in Materials and Methods. BHK cells were labeled with 4 μM C6-NBD-SM for 30 min, washed with buffer medium and imaged on a wide field fluorescence microscope. Mode weights for DMD of rank 5 of this data are shown. The real part of mode weights is shown in left panels (‘Real’), while the imaginary parts are shown in right panels (‘Imag’). **F**, bleach rate fitting using a stretched exponential function with bleaching amplitudes (right panel), time constant (middle panel) and background term (left panel).

**Additional file 13:** Simulated bleach stack. Raw data set 1 used in the analysis shown in Figs. 2 and 3. Photobleaching was simulated using single-exponential decay functions as described in Materials and Methods.

**Additional file 14:** Experimental bleach stack of C. elegans labeled with DHE. Raw data set 2 used in the analysis shown in Figs. 4–7. C. elegans was labeled with DHE, and images were acquired with 0.5 sec acquisition time and without pause as described in Materials and Methods.

**Additional file 15:** Experimental bleach stack of glo-mutant C. elegans labeled with DHE. Raw data set 3 used in the analysis shown in Fig. S5. C. elegans glo-mutant expressing eGFP-rab5 was labeled with DHE, and images were acquired with 1.0 sec acquisition time and without pause as described in Materials and Methods.

**Additional file 16:** Experimental bleach stack of BHKasc cells double-labeled with C6-NBD-SM and Alexa488-Tf. Raw data set 4 used in the analysis shown in Figs. 8 and S12. BHKasc cells were labeled with C6-NBD-SM and Alexa488-TF, and images were acquired with 0.3 sec acquisition time and without pause as described in Materials and Methods.

**Additional file 17:** Experimental bleach stack of BHKasc cells labeled with Alexa488-Tf. Raw data set 5 used in the analysis shown in Fig. S10. BHKasc cells were labeled with Alexa488-TF, and images were acquired with 0.3 sec acquisition time and without pause as described in Materials and Methods.

**Additional file 18:** Experimental bleach stack of BHKasc cells labeled with C6-NBD-SM. Raw data set 6 used in the analysis shown in Fig. S11. BHKasc cells were labeled with C6-NBD-SM, and images were acquired with 0.3 sec acquisition time and without pause as described in Materials and Methods.

## Data Availability

All data analyzed during this study are included in this published article [and its supplementary information files]. The datasets generated and/or analyzed during the current study are also available in the GITHUB repository, (https://github.com/DanielW-alt/Photobleaching).
