# Supplementary figures and images for: Image segmentation and separation of spectrally similar dyes in fluorescence microscopy by dynamic mode decomposition of photobleaching kinetics
Source: BMC Bioinformatics. 2022 Aug 12;23:334. doi: 10.1186/s12859-022-04881-x (PMC9373304; doi:10.1186/s12859-022-04881-x)

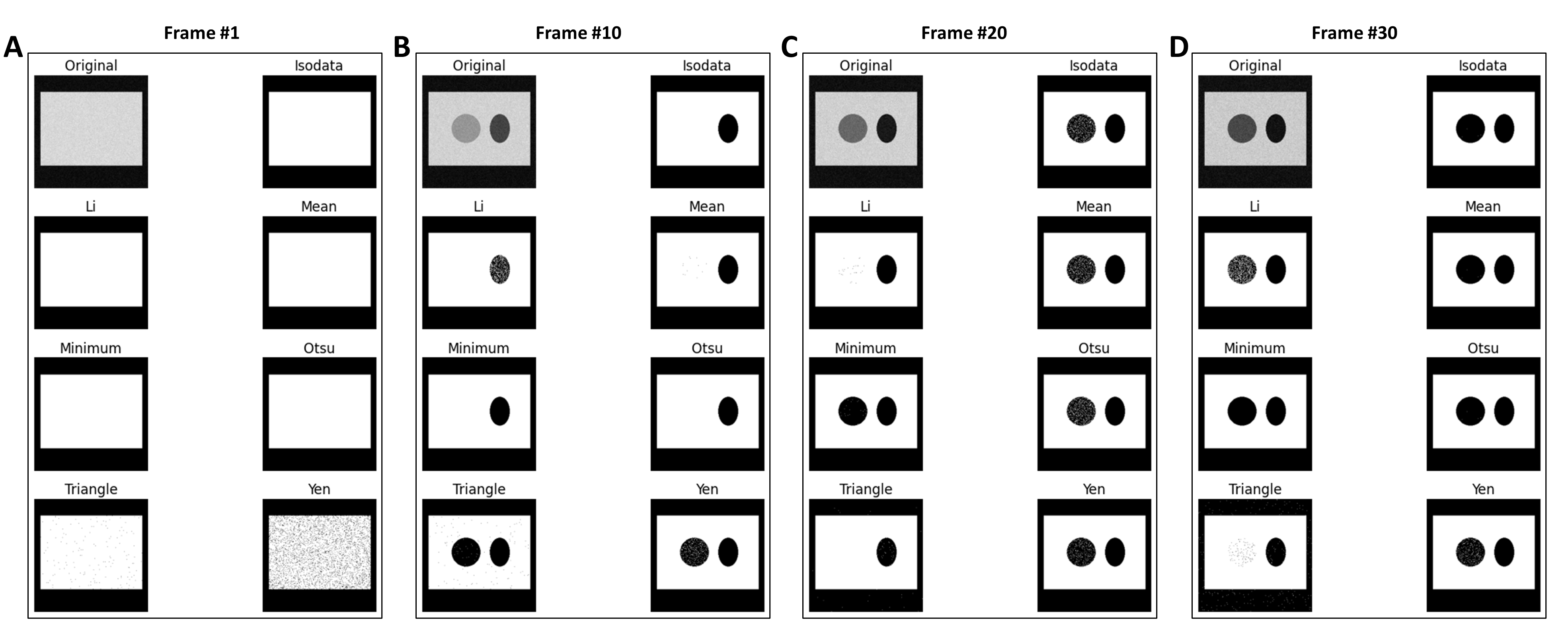

Supplement: Supplementary file 1 — Additional file 1: Fig. S1. Comparison of image thresholding methods for segmentation of synthetic bleach stacks. Several standard thresholding methods, (i.e., Isodata, Li, Mean, Minimum, Otsu, Triangle and Yen method [34]) were assessed in their ability to correctly segment the three image regions of the synthetic bleach stacks. Based on the first image of the stack, all methods could segment the rectangular region from the background but could not dissect the circular and elliptical region (A). Based on the 10th frame of the bleach stack, Isodata, Li, Mean, Minimum and Otsu could segment the rectangular region without the elliptical region, but only the Triangle and Yen method could additionally separate the circular region (B). Similar results were found for the 20th and 30th image frame (C and D and Fig. S2). [file 12859_2022_4881_MOESM1_ESM.png]

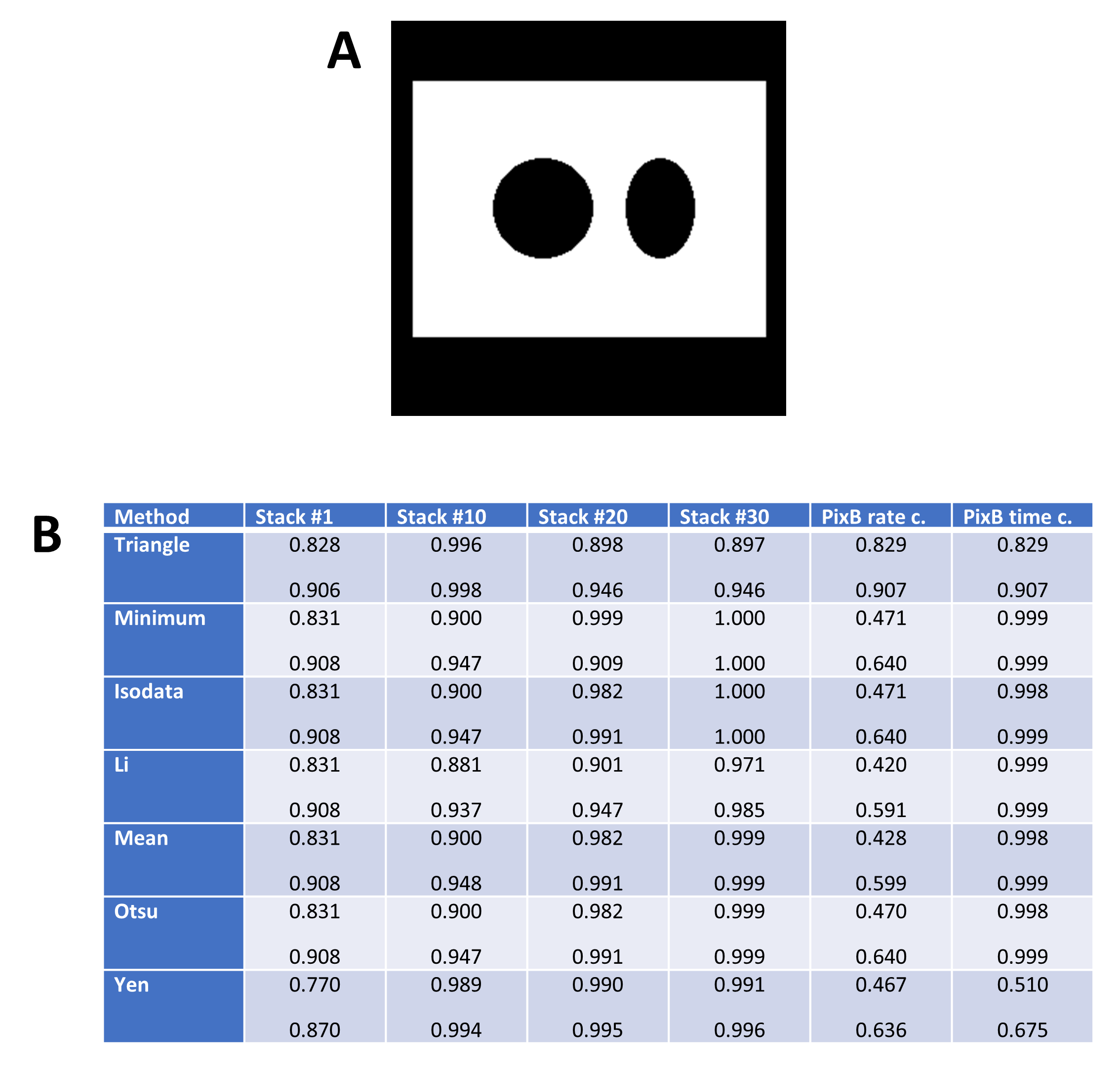

Supplement: Supplementary file 2 — Additional file 2: Fig. S2. Jaccard index and Dice score for image segmentation of rectangular region from the synthetic bleach stack using standard thresholding methods. A, the rectangular region without enclosed circular and elliptical region as ground truth image (white is foreground, black is background). B, Jaccard index, upper rows, and Dice score, lower rows, were calculated for the indicated thresholding methods applied to the 1st, 10th, 20th and 30th frame of the bleach stack as well as for the rate constant and time constant images derived from pixel-wise bleaching analysis in PixBleach relative to the ground truth image. [file 12859_2022_4881_MOESM2_ESM.png]

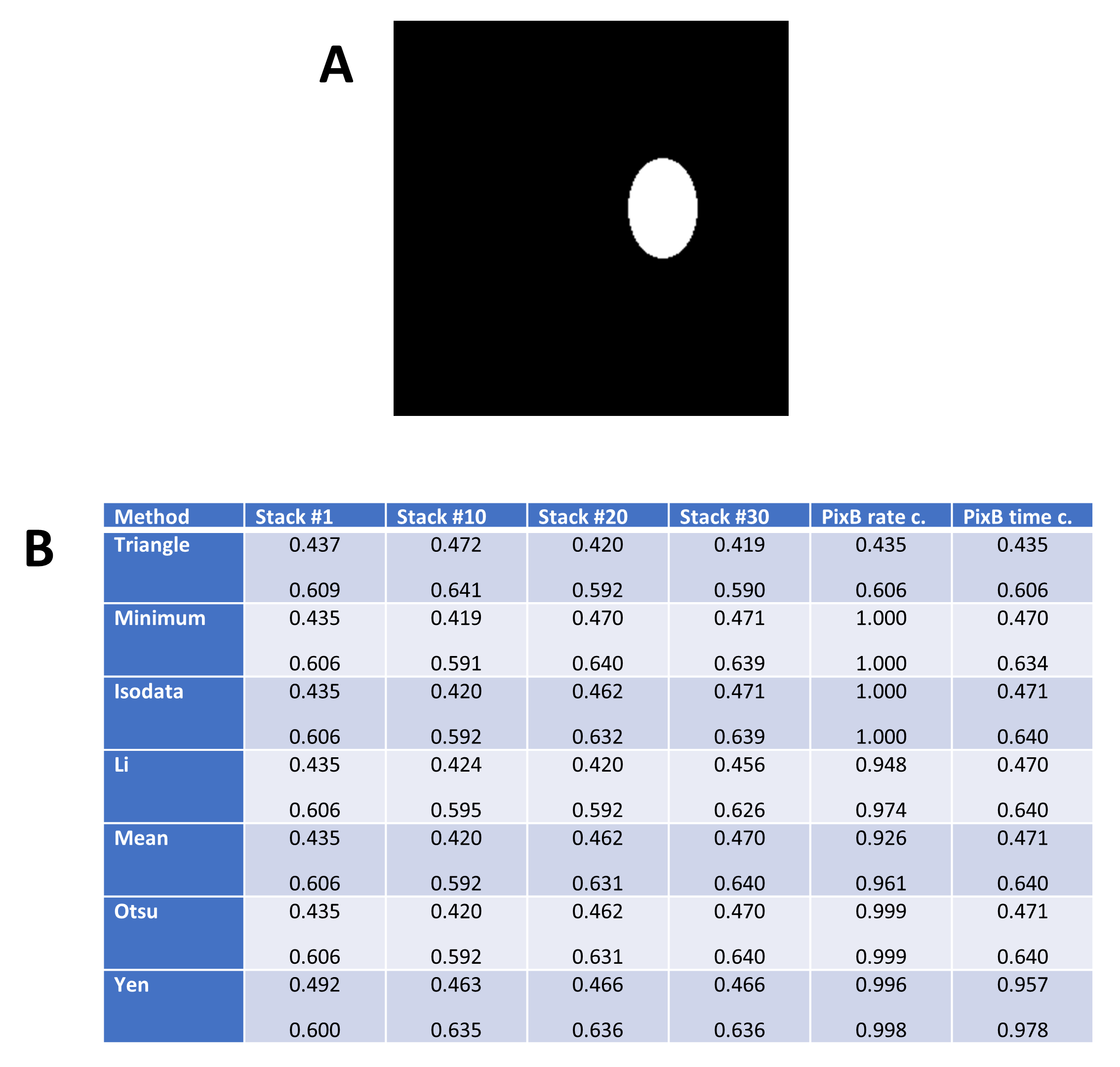

Supplement: Supplementary file 3 — Additional file 3: Fig. S3. Jaccard index and Dice score for image segmentation of elliptical region from the synthetic bleach stack using standard thresholding methods. A, the elliptical region as ground truth image (white is foreground, black is background). B, Jaccard index, upper rows, and Dice score, lower rows, were calculated for the indicated thresholding methods applied to the 1st, 10th, 20th and 30th frame of the bleach stack as well as for the rate constant and time constant images derived from pixel-wise bleaching analysis in PixBleach relative to the ground truth image. [file 12859_2022_4881_MOESM3_ESM.png]

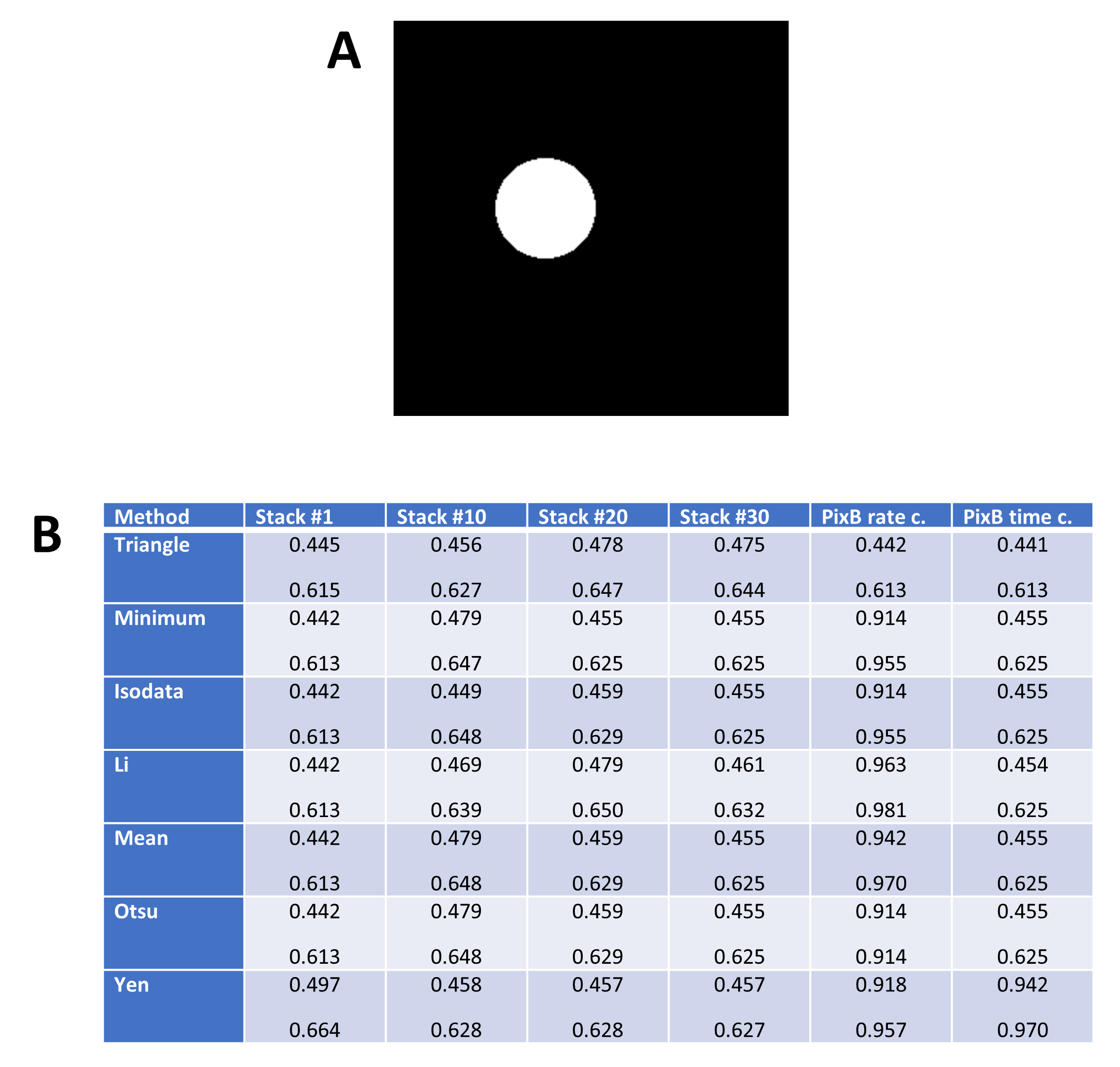

Supplement: Supplementary file 4 — Additional file 4: Fig. S4. Jaccard index and Dice score for image segmentation of circular region from the synthetic bleach stack using standard thresholding methods. A, the circular region as ground truth image (white is foreground, black is background). B, Jaccard index, upper rows, and Dice score, lower rows, were calculated for the indicated thresholding methods applied to the 1st, 10th, 20th and 30th frame of the bleach stack as well as for the rate constant and time constant images derived from pixel-wise bleaching analysis in PixBleach relative to the ground truth image. [file 12859_2022_4881_MOESM4_ESM.png]

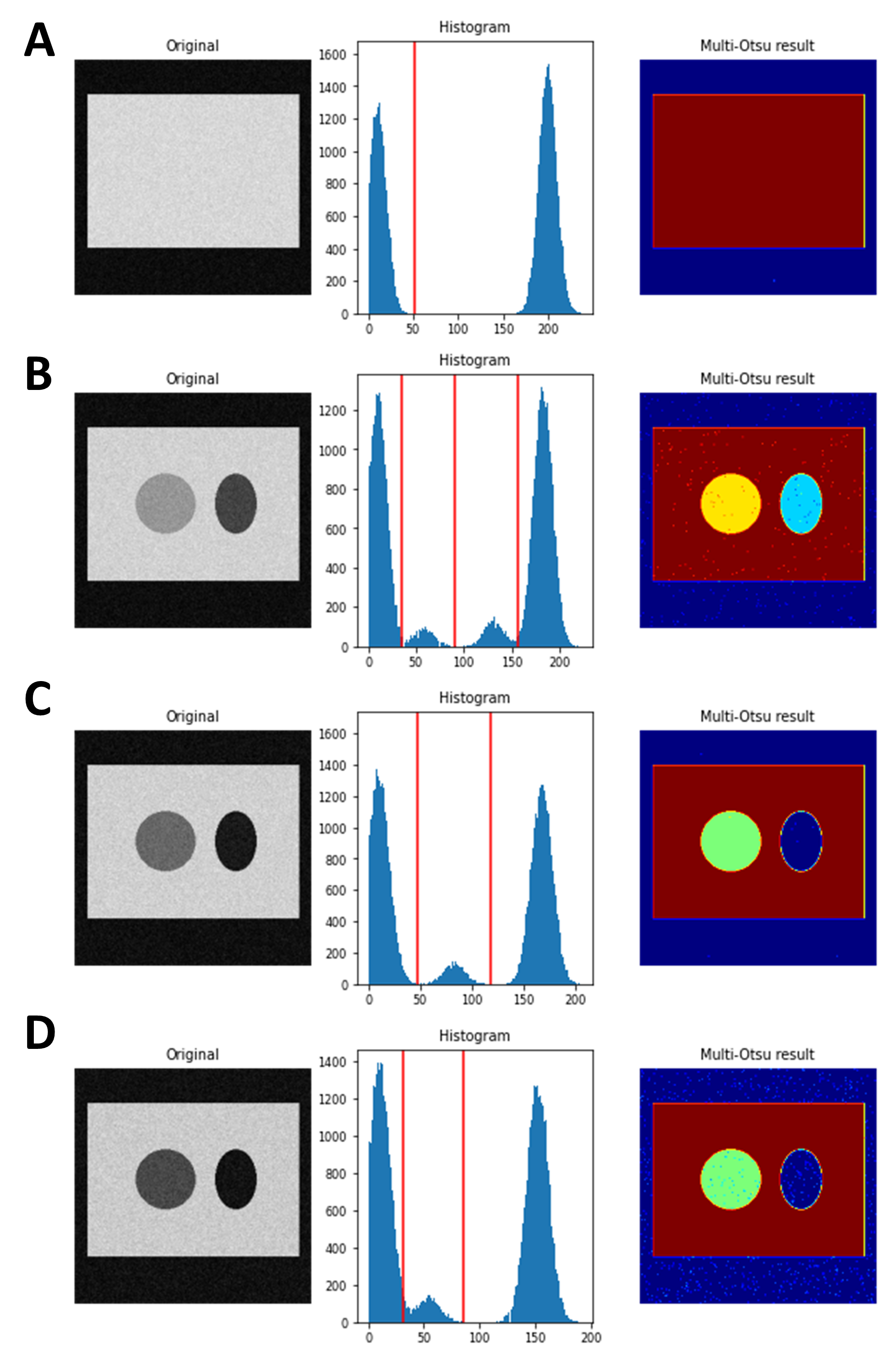

Supplement: Supplementary file 5 — Additional file 5: Fig. S5. Multi-Otsu thresholding of synthetic bleach stack shows varying results depending on which frame is analyzed. Multi-Otsu thresholding was applied to the 1st, 10th, 20th and 30th frame of the bleach stack [38]. For the 1st frame (A), only two regions could be distinguished (brown is foreground, dark blue is background). For the 10th frame (B), four regions could be distinguished (brown, yellow and light blue are foreground of the rectangular, circular and elliptical region, respectively; dark blue is background). For the 20th frame (C) and 30th (D), three regions could be distinguished (brown and light green are foreground of the rectangular and circular region, respectively; dark blue is background). Left panels are original images of the synthetic stack, middle panels are histograms with identified threshold indicated in red, right panels are thresholding results with color-labeled regions. [file 12859_2022_4881_MOESM5_ESM.png]

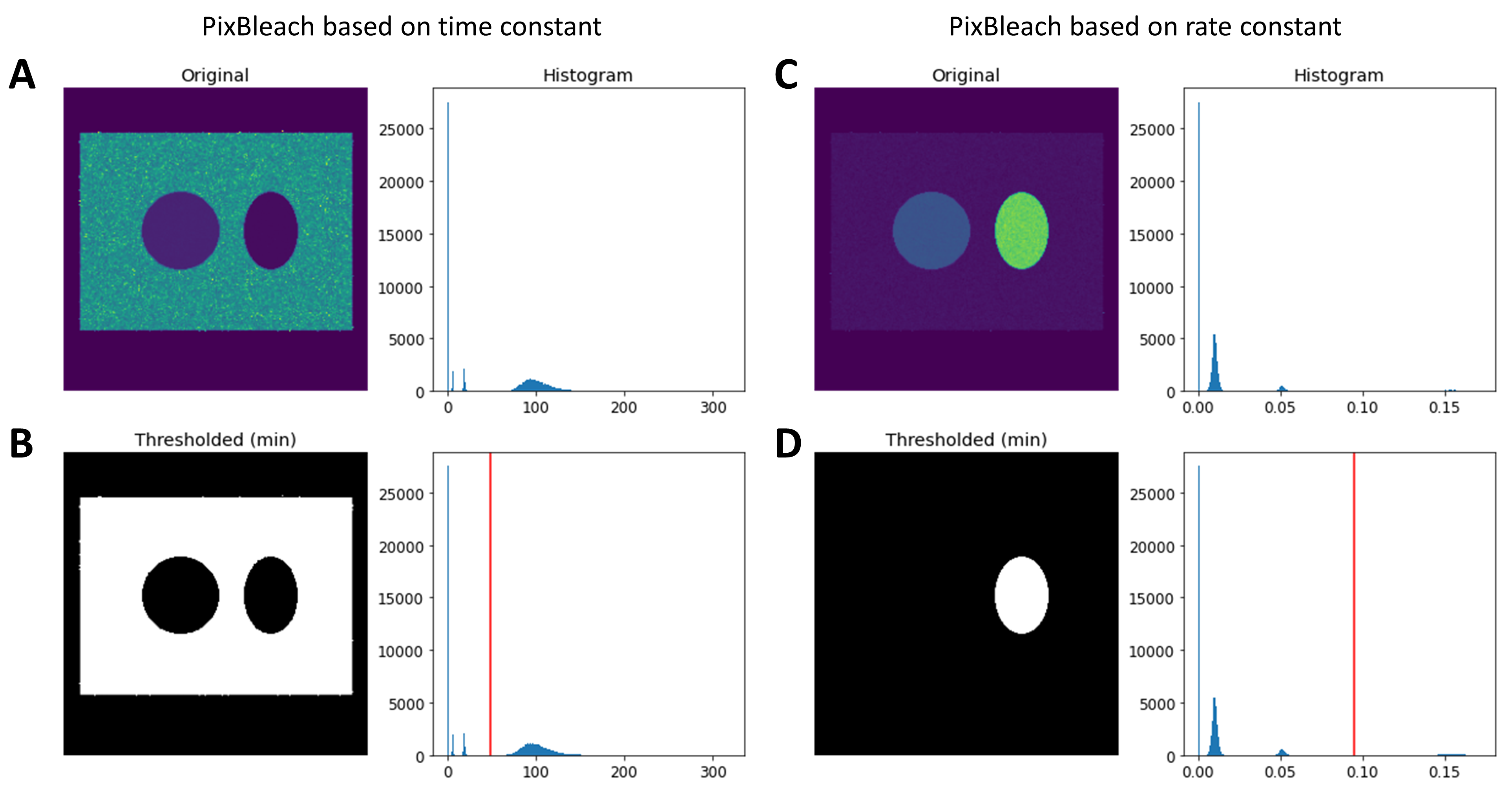

Supplement: Supplementary file 6 — Additional file 6: Fig. S6. Minimum-based thresholding of time and rate constant maps derived from pixel-wise fitting of exponential decay functions to synthetic bleach stack. Pixel-wise bleach rate fitting was applied to the synthetic bleach stack as described in Materials and methods [8]. The resulting time constant image (A and B) and rate constant image (C and D) were segmented using the Minimum threshold method. Left panel in A and C shows the time and rate constant maps, respectively, and right panels show the corresponding histograms. Left panels in B and D show segmentation results with foreground in white and background in black. Right panels in B and D show histograms with identified threshold indicated in red. [file 12859_2022_4881_MOESM6_ESM.png]

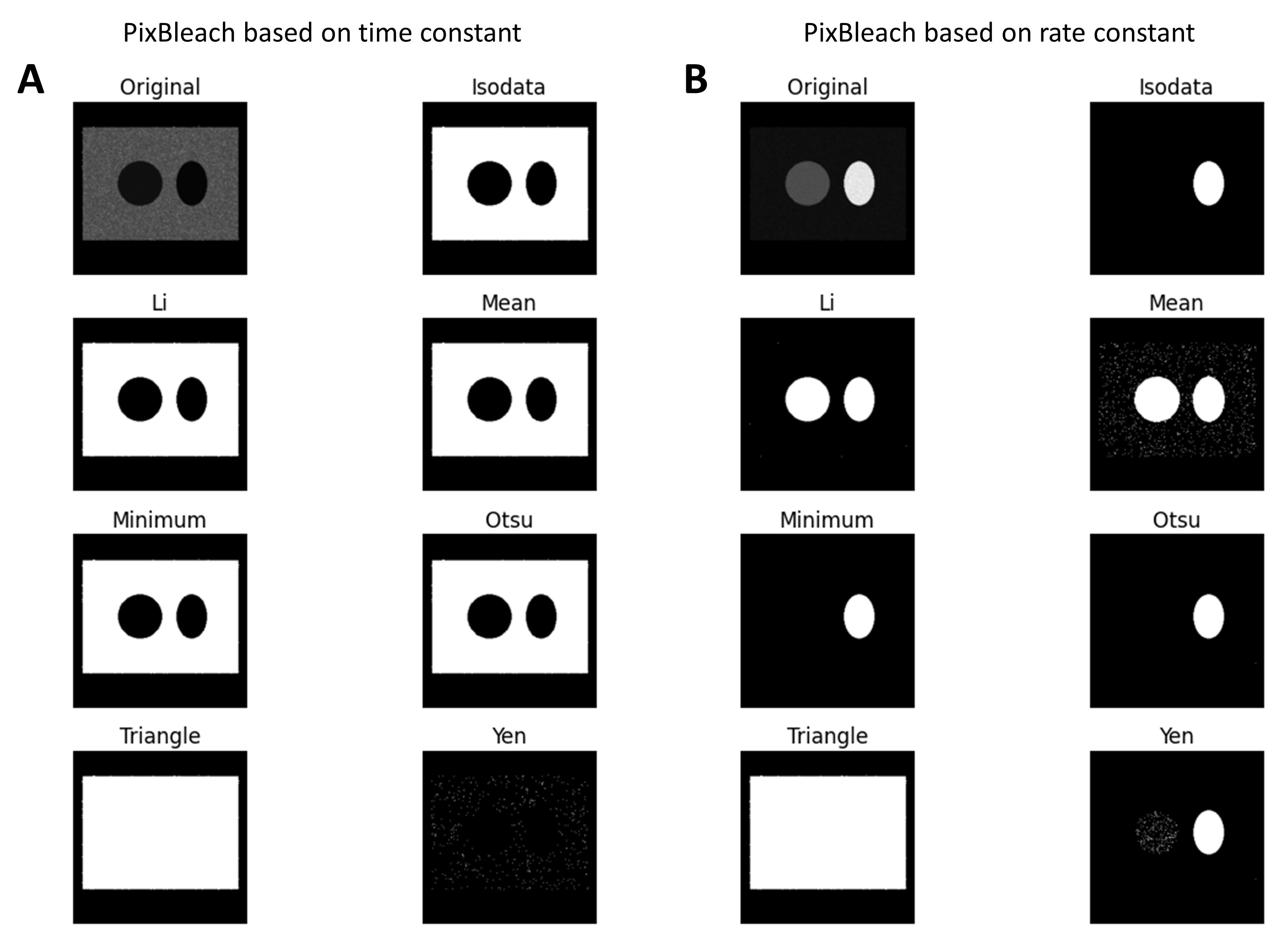

Supplement: Supplementary file 7 — Additional file 7: Fig. S7. Comparison of image thresholding methods for segmentation of time and rate constant maps derived from pixel-wise fitting of exponential decay functions to synthetic bleach stack. Several standard thresholding methods, (i.e., Isodata, Li, Mean, Minimum, Otsu, Triangle and Yen method [34]) were assessed in their ability to correctly segment the three image regions of the time (A) and rate constant maps (B) derived from pixel-wise fitting of exponential decay functions to the synthetic bleach stack. [file 12859_2022_4881_MOESM7_ESM.png]

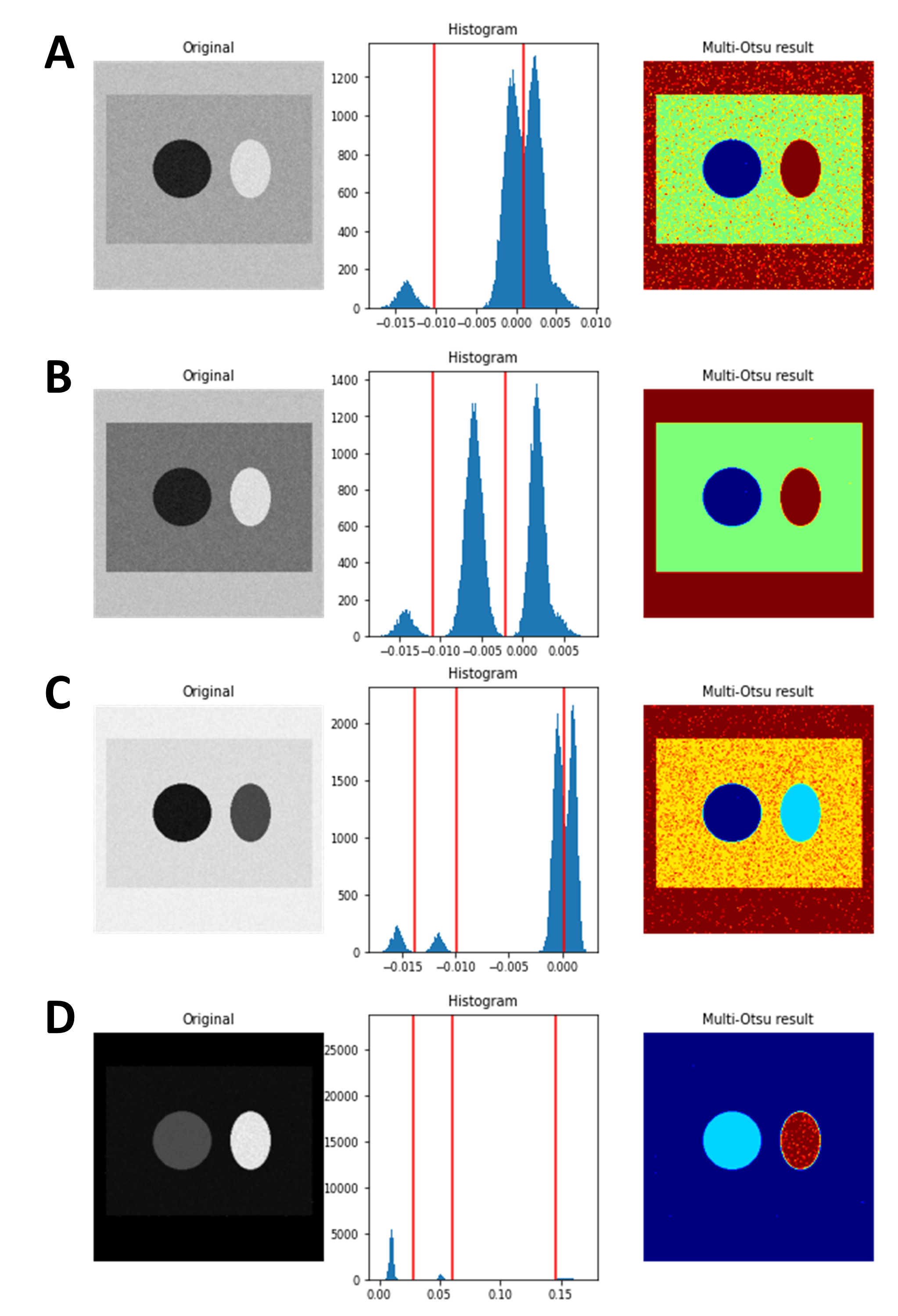

Supplement: Supplementary file 8 — Additional file 8: Fig. S8. Multi-Otsu thresholding of DMD and pixel-wise bleach rate fitting outputs of synthetic bleach stack. Multi-Otsu thresholding was applied to Mode 3 of the DMD (A), to the sum of Mode 1 and 3 (B), to the sum of Mode 2 and 3 (C) and to the rate constant map derived from pixel-wise fitting of exponential decay functions to the synthetic bleach stack (D) [38]. Left panels are the original analyzed images, middle panels are histograms with identified threshold indicated in red, right panels are thresholding results with color-labeled regions. The number of identified regions for each image equals the number of red lines plus one (i.e., three regions in A and B and four regions in C and D). [file 12859_2022_4881_MOESM8_ESM.png]

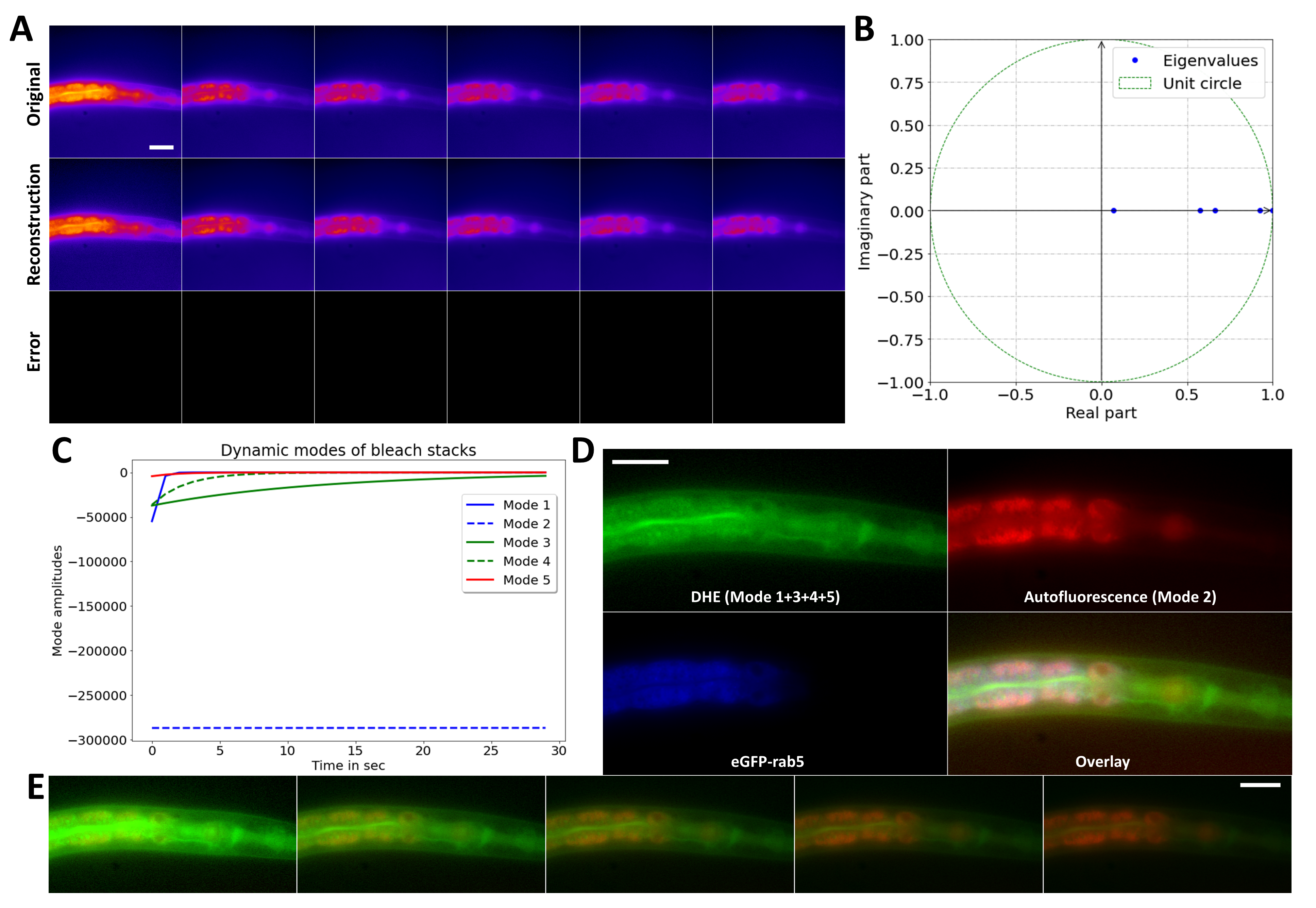

Supplement: Supplementary file 9 — Additional file 9: Fig. S9. Dynamic mode decomposition of fluorescence images of DHE-labeled glo-mutant nematodes with reduced autofluorescence. A, montage of selected images of original (upper row) and reconstructed (lower row) image sequence shown as every fifth image of the corresponding image stack. The lowest panel shows the absolute error between original and reconstructed image sequence with same intensity scaling. First five eigenvalues of space-time matrix of experimental image sequence (B) and corresponding dynamic modes (C) determined by DMD. Sum of dynamic mode 1, 3, 4 and 5 resemble total DHE fluorescence (upper left panel in green in D). Mode 2 resembles cellular autofluorescence of nematodes (upper right panel in red in D). These worms also express eGFP-rab5 as marker for early and recycling endosomes in their intestine (lower left panel in blue in D). Some of the DHE and autofluorescence signal overlap with eGFP-rab5 in the intestine (lower right panel in D). E, color overlay of mode decomposition with dynamic mode 2 resembling autofluorescence in red and sum of mode 1, 3, 4 and 5 representing DHE fluorescence in green. Bar, 20 μm. [file 12859_2022_4881_MOESM9_ESM.png]

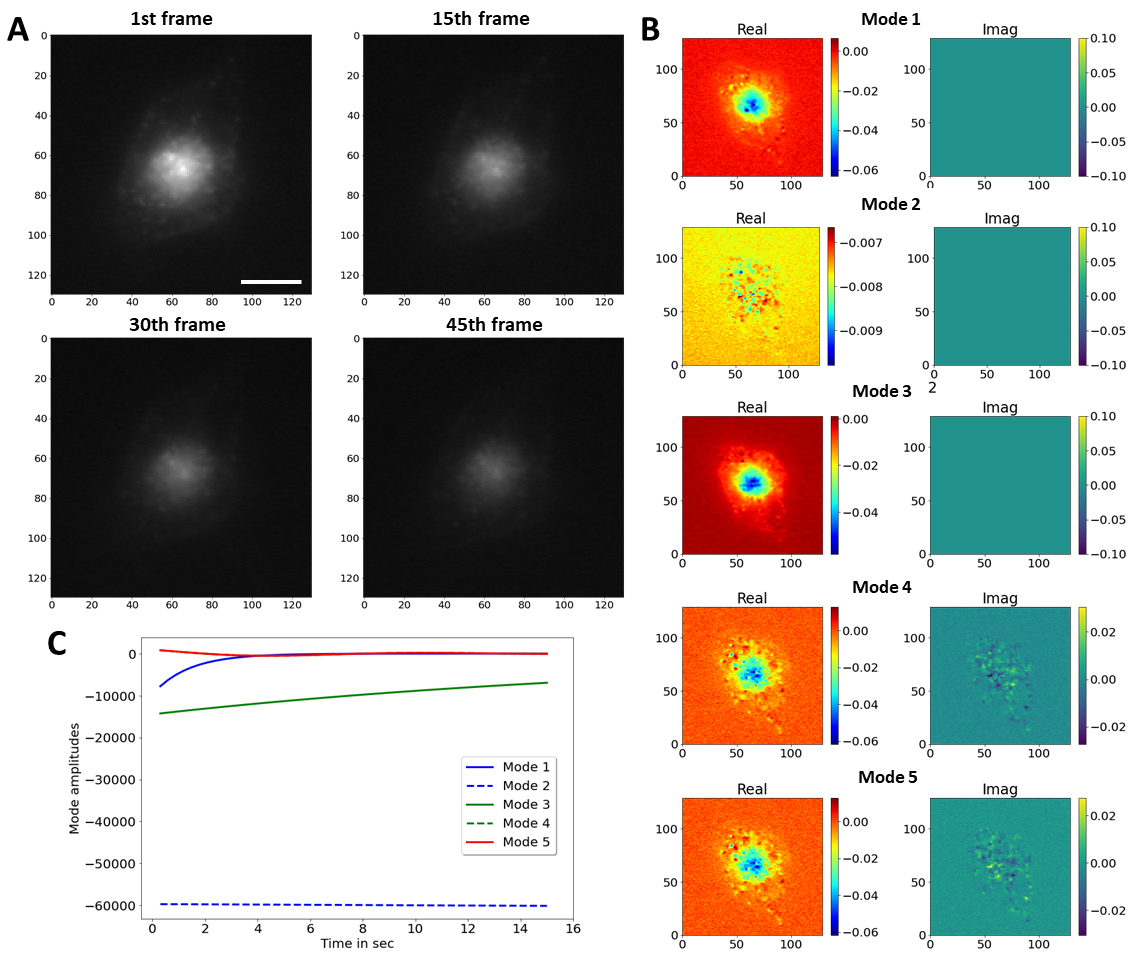

Supplement: Supplementary file 10 — Additional file 10: Fig. S10. Dynamic mode decomposition of image stacks containing Alexa488-Tf labeled cells. BHK cells were labeled with 20 μg/ml Alexa488-Tf for 30 min, washed with buffer medium and imaged on a wide field fluorescence microscope. A, selected frames of an image stack acquired with 0.3 sec acquisition time and without pause. Images are identically scaled; bar 10 μm. B, C, DMD of this image stack using a rank-5 approximation to the full transfer matrix. B, mode weights and C, mode amplitudes as function of time. [file 12859_2022_4881_MOESM10_ESM.png]

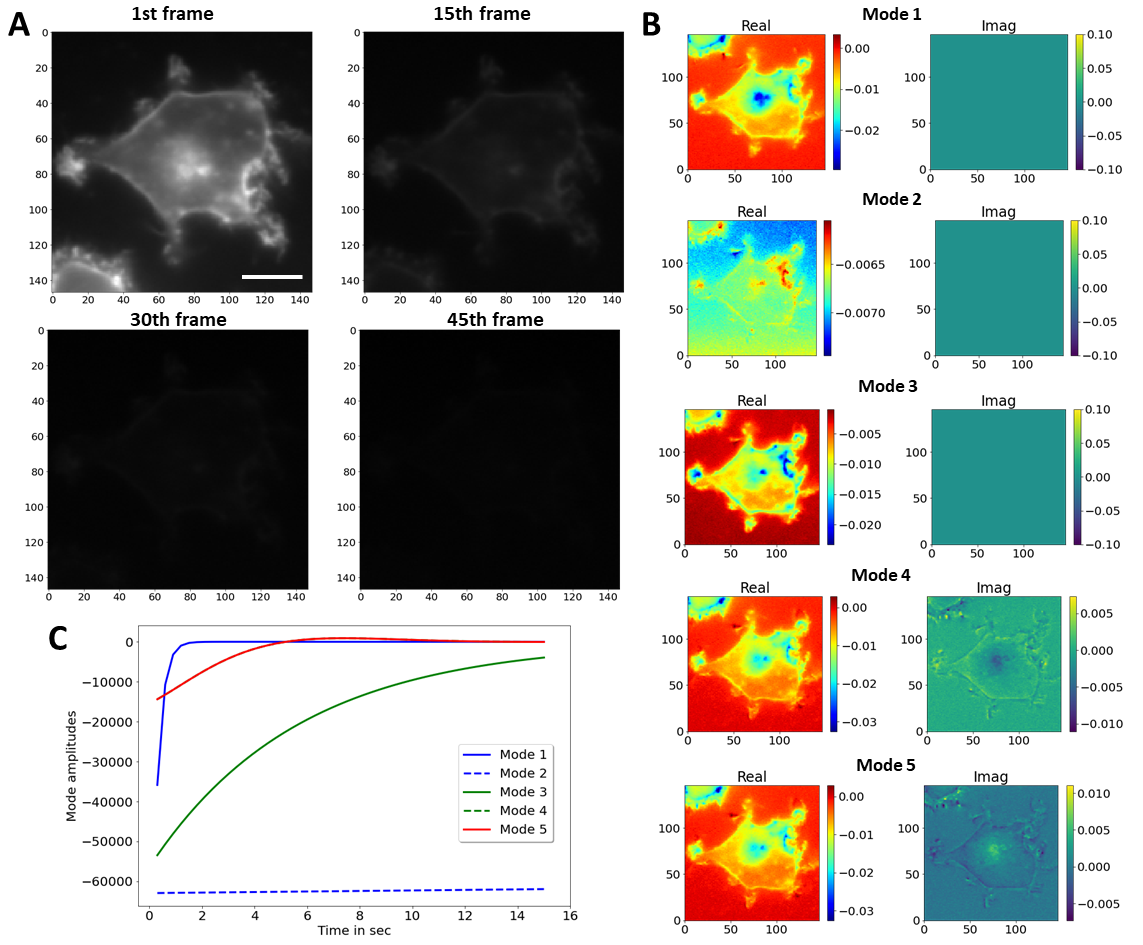

Supplement: Supplementary file 11 — Additional file 11: Fig. S11. Dynamic mode decomposition of image stacks containing C6-NBD-SM labeled cells. BHK cells were labeled with 4 μM C6-NBD-SM for 30 min, washed with buffer medium and imaged on a wide field fluorescence microscope. A, selected frames of an image stack acquired with 0.3 sec acquisition time and without pause. Images are identically scaled; bar 10 μm. B, C, DMD of this image stack using a rank-5 approximation to the full transfer matrix. B, mode weights and C, mode amplitudes as function of time. [file 12859_2022_4881_MOESM11_ESM.png]

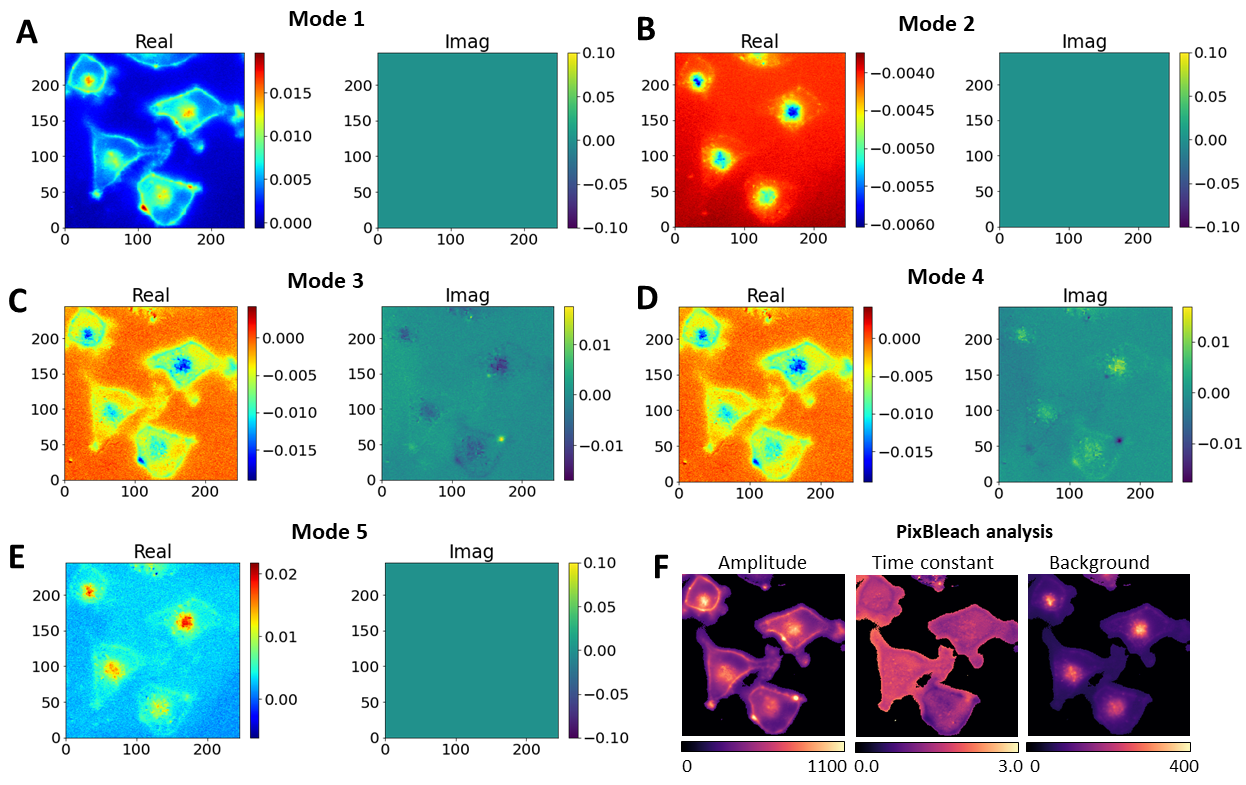

Supplement: Supplementary file 12 — Additional file 12: Fig. S12. Mode weights for DMD of image stacks of BHK cells labeled with C6-NBD-SM and Alexa488-Tf. BHK cells were labeled with 4 μM C6-NBD-SM and with 20 μg/ml Alexa488-Tf, both emitting in green, as described in Materials and Methods. BHK cells were labeled with 4 μM C6-NBD-SM for 30 min, washed with buffer medium and imaged on a wide field fluorescence microscope. Mode weights for DMD of rank 5 of this data are shown. The real part of mode weights is shown in left panels (‘Real’), while the imaginary parts are shown in right panels (‘Imag’). F, bleach rate fitting using a stretched exponential function with bleaching amplitudes (right panel), time constant (middle panel) and background term (left panel). [file 12859_2022_4881_MOESM12_ESM.png]

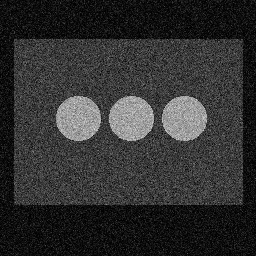

Supplement: Supplementary file 13 — Additional file 13: Simulated bleach stack. Raw data set 1 used in the analysis shown in Figs. 2 and 3. Photobleaching was simulated using single-exponential decay functions as described in Materials and Methods. [file 12859_2022_4881_MOESM13_ESM.tif]

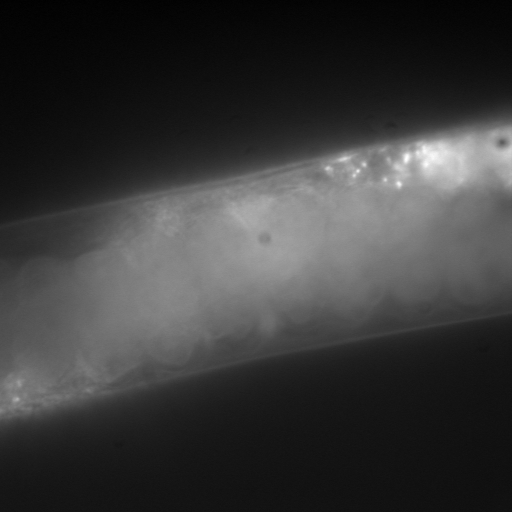

Supplement: Supplementary file 14 — Additional file 14: Experimental bleach stack of C. elegans labeled with DHE. Raw data set 2 used in the analysis shown in Figs. 4–7. C. elegans was labeled with DHE, and images were acquired with 0.5 sec acquisition time and without pause as described in Materials and Methods. [file 12859_2022_4881_MOESM14_ESM.tif]

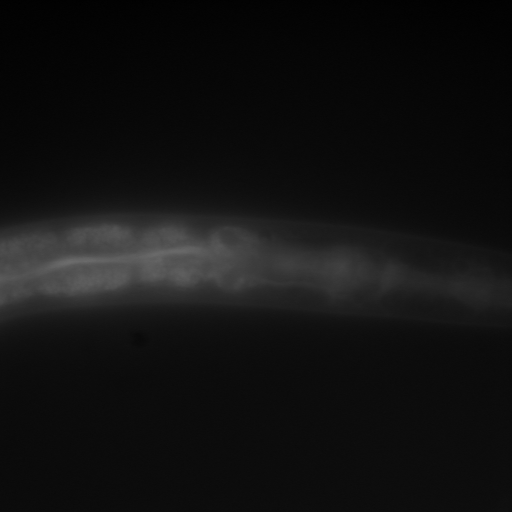

Supplement: Supplementary file 15 — Additional file 15: Experimental bleach stack of glo-mutant C. elegans labeled with DHE. Raw data set 3 used in the analysis shown in Fig. S5. C. elegans glo-mutant expressing eGFP-rab5 was labeled with DHE, and images were acquired with 1.0 sec acquisition time and without pause as described in Materials and Methods. [file 12859_2022_4881_MOESM15_ESM.tif]

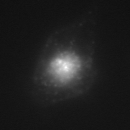

Supplement: Supplementary file 17 — Additional file 17: Experimental bleach stack of BHKasc cells labeled with Alexa488-Tf. Raw data set 5 used in the analysis shown in Fig. S10. BHKasc cells were labeled with Alexa488-TF, and images were acquired with 0.3 sec acquisition time and without pause as described in Materials and Methods. [file 12859_2022_4881_MOESM17_ESM.tif]

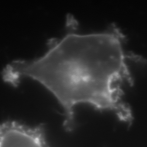

Supplement: Supplementary file 18 — Additional file 18: Experimental bleach stack of BHKasc cells labeled with C6-NBD-SM. Raw data set 6 used in the analysis shown in Fig. S11. BHKasc cells were labeled with C6-NBD-SM, and images were acquired with 0.3 sec acquisition time and without pause as described in Materials and Methods. [file 12859_2022_4881_MOESM18_ESM.tif]
